# Histopathological-based brain tumor grading using 2D-3D multi-modal CNN-transformer combined with stacking classifiers

**DOI:** 10.1038/s41598-025-11754-9

**Published:** 2025-07-30

**Authors:** Naira Elazab, Fahmi Khalifa, Wael Gab Allah, Mohammed Elmogy

**Affiliations:** 1 Information Technology Department, Faculty of Computers and Information, Mansoura University, Mansoura, 35516 Egypt; 2 Electronics and Communications Engineering, Faculty of Engineering, Mansoura University, Mansoura, 35516 Egypt

**Keywords:** Brain tumor grading, Histopathological image analysis, Hybrid deep learning architecture, Vision transformer, 2D-3D convolutional neural network, Stacking classifiers, Computer science, Electrical and electronic engineering

## Abstract

Reliability in diagnosing and treating brain tumors depends on the accurate grading of histopathological images. However, limited scalability, adaptability, and interpretability challenge current methods for frequently grading brain tumors to accurately capture complex spatial relationships in histopathological images. This highlights the need for new approaches to overcome these shortcomings. This paper proposes a comprehensive hybrid learning architecture for brain tumor grading. Our pipeline uses complementary feature extraction techniques to capture domain-specific knowledge related to brain tumor morphology, such as texture and intensity patterns. An efficient method of learning hierarchical patterns within the tissue is the 2D-3D hybrid convolution neural network (CNN), which extracts contextual and spatial features. A vision transformer (ViT) additionally learns global relationships between image regions by concentrating on high-level semantic representations from image patches. Finally, a stacking ensemble machine learning classifier is fed concatenated features, allowing it to take advantage of the individual model’s strengths and possibly enhance generalization. Our model’s performance is evaluated using two publicly accessible datasets: TCGA and DeepHisto. Extensive experiments with ablation studies and cross-dataset evaluation validate the model’s effectiveness, demonstrating significant gains in accuracy, precision, and specificity using cross-validation scenarios. In total, our brain tumor grading model outperforms existing methods, achieving an average accuracy, precision, and specificity of 97.1%, 97.1%, and 97.0%, respectively, on the TCGA dataset, and 95%, 94%, and 95% on DeepHisto dataset. Reported results demonstrate how the suggested architecture, which blends deep learning (DL) with domain expertise, can achieve reliable and accurate brain tumor grading.

## Introduction

Brain tumors are a serious type of brain disease distinguished by uncontrolled cell growth within the skull. These tumors can be classified as primary or secondary. The former refers to the brain tumors originating within the brain and account for approximately 70% of all cases. In contrast, the latter refers to those spread from other parts of the body^1^. Gliomas, the most common and aggressive type of brain tumor, develop from glial cells, which support and protect neurons^2^. Early and precise classification of gliomas is critical for successful treatment. This information informs treatment plans and significantly impacts patient survival rates^3^. Although neuropathologists currently use microscopic analysis of biopsies to diagnose gliomas, this process is laborious, subjective, and prone to inconsistency. This is especially worrying in the current era of rapidly developing cancer treatment options, where patient survival depends on an accurate and timely diagnosis^4,5^.

In order to expedite and enhance glioma diagnosis and improve patient outcomes, new techniques are desperately needed^6^. Recent advancements in computational techniques using machine learning (ML) and deep learning (DL) methods have surged dramatically in helping with the above-mentioned problems in various application domains. This advancement in artificial intelligence (AI)-powered diagnostics holds great promise for improving patient outcomes by allowing for earlier detection and more effective treatment plans^7^.

Recent literature documents that advances in DL have accelerated the development of automated glioma classification. Convolutional neural networks (CNNs) have shown promise in this field, excelling at extracting local features to distinguish specific cellular patterns characteristic of various glioma types^8,9^. However, CNNs prioritize pixel-level information while ignoring the image’s broader context. This limitation can be problematic when analyzing complex medical images, such as brain biopsies, where understanding the global relationships between features is critical for accurate classification^10,11^. Vision transformers (ViT), another robust DL architecture, overcome this limitation by excelling at capturing global features. Their ability to analyze the entire image at once and identify long-term dependencies between features makes them ideal for tasks that require a comprehensive understanding. Conversely, transformers can have difficulty extracting the fine-grained details and localized patterns required for glioma classification^12^.

Recognizing the strengths and weaknesses of both CNNs and transformers, this paper proposes a hybrid architecture that takes advantage of their complementary capabilities. Our design aims to overcome the limitations of individual models and achieve enhanced accuracy in glioma classification. This advancement has the potential to significantly improve patient outcomes by allowing for earlier and more precise diagnoses, resulting in better-informed treatment decisions.

This work aims to enhance the characterization of brain tumors through DL techniques when combined with handcrafted features, thus enhancing feature representation. The proposed model is built on a unique combination of handcrafted features and DL techniques. Handcrafted features extracted from histopathological images capture important morphological and textural information that conventional automated methods frequently miss. When these features are combined with the cutting-edge capabilities of hybrid 2D-3D CNNs and ViT, the model gains an unrivaled ability to comprehend the nuanced details and the larger spatial contexts within the images. The 2D CNN layers are excellent at identifying patterns and features in individual image slices.

In contrast, the 3D CNN layers delve deeper into spatial relationships, capturing the information required for accurate brain tumor analysis. Our approach is centered on using a multiple-stacking classifier. This advanced ensemble technique combines the outputs of the various model components, synthesizing them with a meta-classifier to produce a final decision. This strategy takes advantage of the various perspectives provided by each component and significantly improves the model’s robustness and reliability. The proposed hybrid framework makes several significant contributions to the field of brain tumor computational pathology, which can be summarized as follows:

- Incorporating handcrafted features improves the model’s ability to capture morphological and textural information from histopathological images, providing valuable insights from experts. ViT improves diagnostic accuracy by focusing on the most informative regions in images, regardless of their spatial location. Combining 2D and 3D CNN architectures allows for comprehensive analysis of brain tumor images, capturing both local patterns and spatial relationships.
- Multiple stacking ensemble classifier combines outputs from multiple models to improve robustness and reliability by leveraging individual strengths and mitigating weaknesses.
- Comprehensive spatial and feature representation uses handcrafted features, 2D-3D CNNs, and ViT to provide a holistic representation of spatial relationships and morphological features in histopathological images. This provides valuable insights for brain tumor classification.
- Improved Diagnostic Accuracy and Reliability: Combining multiple model components and ensemble techniques leads to higher diagnostic accuracy and reliability, setting a new standard in brain tumor computational pathology.
- Comprehensive evaluation and ablation studies: Two independent datasets, The Cancer Genome Atlas (TCGA) and DeepHisto, are used in a thorough evaluation. To further confirm the model’s capacity to generalize to new data, it is also tested on a different dataset after being trained on the first one.

Current AI models for brain tumor grading face a critical trade-off: CNNs excel at local texture analysis (e.g., tumor margins) but lack global context, while Transformers model long-range dependencies (e.g., necrosis distribution) but are computationally prohibitive for high-resolution histopathology. To bridge this gap, we propose a hybrid 2D-3D CNN-Transformer architecture. The 3D CNN processes multi-scale stain decompositions (Hematoxylin/Eosin) to capture spatial-spectral patterns, while the Transformer focuses on diagnostically critical regions via self-attention. This synergy enables precise, interpretable grading while maintaining computational efficiency.

The rest of this paper is organized into four consecutive sections. Section “Related work” examines the current literature and methodologies developed for brain tumor computational pathology, identifying gaps and opportunities for innovation. Section “Methodology” describes the proposed hybrid architecture and its parameter settings in detail. Section “Experimental results” presents quantitative and qualitative findings from experiments on the TCGA and DeepHisto datasets, validated using various ablation schemes and evaluation criteria. Finally, Section “Conclusion” summarizes key findings, discusses implications, and proposes avenues for future research.

## Related work

The classification of brain tumors from histopathological images has evolved significantly with the advancement of deep learning, particularly in the context of glioma diagnosis and grading^13^. Recent literature highlights three dominant research themes: conventional deep learning with CNNs, hybrid fusion of handcrafted and deep features, and transformer-based architectures with attention mechanisms. While these directions have shown promising performance, limitations remain in adaptability, interpretability, and fusion efficacy – motivating the need for more robust and intelligent frameworks.

### Deep learning-based feature extraction and classification

Deep convolutional neural networks (CNNs) have emerged as dominant tools for extracting discriminative features from histopathological images. Early works like Yonekura et al.^14,15^ utilized CNNs on TCGA-provided GBM images to automate disease stage classification, achieving promising accuracies. This foundation has been extended in broader surveys, such as Shirazi et al.^16^, who reviewed CNN applications in brain cancer diagnosis across histology, MRI, and CT modalities, and emphasized CNNs’ potential in personalized care.

More complex pipelines have emerged, combining real-time analysis and deep learning. For instance, Hollon et al.^17^ proposed a CNN integrated with stimulated Raman histology (SRH) to enable bedside diagnosis within seconds–achieving diagnostic accuracy comparable to pathologists. Similarly, Zadeh et al. ^18^ introduced DeepSurvNet to classify patient survival groups based on histological features, reinforcing the predictive capacity of CNN-based models for survival analysis.

### Hybrid feature engineering and fusion strategies

To overcome single-modality limitations, researchers developed hybrid approaches integrating diverse data dimensions. Molecular-cellular fusion was pioneered by Pei et al.^19^, who combined WHO molecular criteria with cellularity features in DNN classifiers (93.8% glioma accuracy). Handcrafted feature ensembles saw significant innovation through Elazab et al.^20^ fused GLCM, LBP, and multi-LBGLCM features (95.8% accuracy). Texture-based methods further enriched this paradigm: Cheung et al.^21^ extracted 33 GLCM/GLRLM features for SVM-based GBM detection (93.5% accuracy on local cohorts), Amin et al.^22^ combined Gabor wavelets with HOG/SFBTA for unsupervised tumor subregion segmentation, and Mohan & Monicasubashini^23^ optimized computational efficiency through tile-based FLBP/Gabor analysis (93.5% accuracy via SVM). Ensemble learning improved robustness through Archana et al.^24^ bagged KNN with U-Net segmentation and Balamurugan & Gnanamanoharan^25^ LuNet classifier enhanced by FCM-GMM segmentation. These innovations demonstrate that strategic fusion of complementary data modalities consistently enhances diagnostic precision.

### Transformer-based models and attention mechanisms

ViTs represent a paradigm shift in whole-slide image (WSI) analysis. Pure ViT architectures like Li et al.^26^ ViT-WSI achieved unprecedented 96% AUC for molecular marker prediction while offering interpretability through gradient-based attribution. Hybrid frameworks advanced further with Zhou et al.^27^ ResNet-ViT network (ASI-DBNet), which reached 95.24% accuracy via adaptive sparse interaction blocks that optimize local-global feature balance. Multi-scale systems addressed resolution challenges through Liu et al.^28^ MultiGeneNet, fusing coarse/fine-grained features via bilinear pooling (83.5% accuracy). Hardware-optimized solutions culminated in Ma et al.^29^ HAS-Bt system, employing pipeline-structured MIL on 1.3M+ patches (94% accuracy) with integrated diagnostic workflows. This architectural evolution highlights how transformers overcome CNN limitations in long-range feature dependency modeling while introducing new computational demands.

Zhang et al.^30^ developed a hybrid ResNet-ViT pipeline for glioma region segmentation and classification, with accuracy reaching 91.7%, while Aboussaleh et al.^31^ utilized multi-head cross-attention capsules to model spatial and contextual hierarchies in glioma WSI patches, reporting 95.6% accuracy. These transformer-based models demonstrate strong performance and better visual interpretability via attention maps. However, they often lack flexible fusion mechanisms when combining features from different sources and rarely integrate global optimization in their pipeline.

Recent advancements such as Global Contrast-Masked Autoencoders (GCMAEs) have further pushed the boundaries of unsupervised representation learning in pathology. Quan et al.^32^ demonstrated that GCMAEs can extract semantically rich and class-discriminative features without requiring dense annotations, offering an efficient and scalable alternative to traditional supervised pipelines. To address data scarcity challenges in histopathological image classification, Quan et al.^33^ proposed a Dual-Channel Prototype Network (DCPN) designed for few-shot pathology tasks, which effectively leverages both global and localized feature representations to enhance classification accuracy under limited supervision scenarios.

Various studies have been proposed to tackle brain tumor classification using pathological data, and Table 1 introduces a summary of recent studies highlighting methodologies, performance metrics, and limitations for brain tumors. Despite the fact that many techniques have been developed with document success, there are a few issues that need to be resolved.

Despite significant advancements, several critical limitations remain unaddressed in current histopathological image analysis frameworks. First, a notable generalization gap is often observed when models trained on large public datasets such as TCGA are applied to local clinical cohorts–for example, Cheung et al. reported a sensitivity drop to 86.9% under such conditions. Second, the computational demands of whole slide image (WSI) analysis continue to hinder real-time diagnostic deployment in clinical settings. Third, many high-performing deep learning models sacrifice interpretability, limiting their clinical acceptance. Recent efforts, such as Ghosal et al.’s integration of multi-modal radiomic and deep learning features, and Hu et al.’s use of conflict-aware transformer-based architectures, highlight a promising shift toward more adaptive and dynamic solutions. In line with these directions, our proposed framework addresses these challenges by incorporating lightweight cross-attention modules and resolution-adaptive mechanisms designed to enhance generalizability, efficiency, and interpretability.

**Table 1:** The summary of the state-of-the-art methodologies, performance metrics, and brain tumor histopathological image analysis limitations.

Study	Method	Performance metrics	Limitations
Yonekura et al.^14^	Deep CNN	96.5% avg. classification accuracy	Limited data, single-center study
Yonekura et al.^15^	Deep CNN	96.5% avg. classification accuracy (deeper network), 98.0% accuracy (other networks)	Limited data, single-center study
Hollon et al.^17^	Deep CNNs on SRH images	94.6% accuracy (comparable to pathologists)	Requires further validation, limited data on generalizability
Zadeh et al.^18^	Deep CNNs for survival rate prediction	0.99,0.8 precision for survival rate classification	Limited data, single-center study
Pei et al.^19^	Deep Neural Network with cellularity features and molecular data	93.81% accuracy (LGG vs. HGG), 73.95% accuracy (LGG II vs. LGG III)	Limited data for LGG grading, requires access to molecular data
Ezuma^34^	Deep Learning (DenseNet-201) and Non-Deep Learning Classifiers	82% accuracy (DenseNet-201), 83.8% accuracy (ANN)	Lower accuracy compared to other studies
Cheung et al.^21^	Machine Learning with handcrafted features	93.5% accuracy, 86.95% sensitivity	Relies on handcrafted features, may not capture full image complexity
Ma et al.^29^	Pipeline-structured multiple instance learning (pMIL) with Deep Learning	94% accuracy	Requires further validation on external datasets
Mohan and Monicasubashini^23^	Machine Learning with various feature descriptors and classifiers	93.5% accuracy (SVM)	Limited analysis of feature importance
Elazab et al.^20^	Combining GLCM, GLRLM, color moment and RSHD techniques for feature extraction	95.% accuracy	High-level semantic features (e.g., deep learning-based representations) are not explored.

A significant obstacle is that a few large and well-annotated datasets are available. Thus, literature models can be applied less to real-world situations due to overfitting caused by this lack of data. Complex DL model training can be computationally expensive and time-consuming. This is another drawback. Furthermore, handmade feature extraction is used in some studies, which can be laborious and subjective for the researchers. However, the intrinsic heterogeneity of brain tumors, where differences in staining intensity and cell morphology can make accurate classification difficult and may make even the most sophisticated DL architectures unreliable. It is also challenging to comprehend how the models determine their classifications, a concern raised by a few studies regarding interpretability.

Several techniques are used in our suggested model to overcome these drawbacks. We overcome the problem of insufficient training data by employing a patch-based methodology. We divided large slide images into more manageable, smaller patches. This lowers the computational requirements and enables us to train the model on a substantially larger effective dataset. To capture global image context, our model combines a ViT with CNNs for local detail extraction. This enables the intricate tissue structures to teach the model a wider range of features. We address the model’s generalizability by training the model on two separate datasets. This guarantees that the model works well on unseen data and reduces the chance of overfitting. Our model combines these strategies to better classify brain tumors from histopathological images with superior robustness, accuracy, and generalizability.

## Methodology

**Fig. 1:**
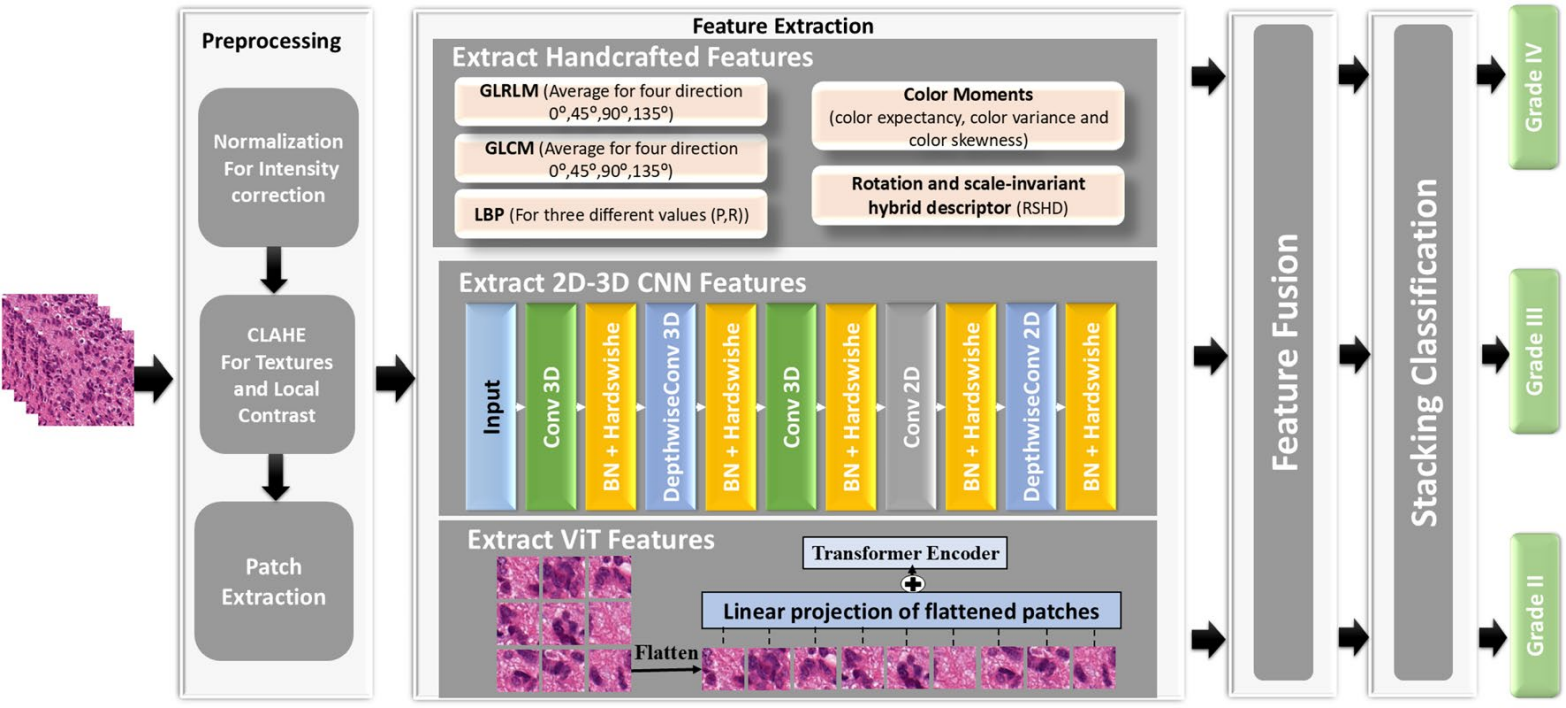
The proposed framework for grading brain tumor using hybrid feature extraction model.

Three main steps are involved in the proposed framework for classifying glioma brain tumors. The detailed proposed framework is presented in Fig. 1. At first, a preprocessing stage is employed to ensure consistency and quality of images. Standard methods like intensity normalization and image patching may be used to prepare the histopathological images for further analysis.

Our methodology revolves around the second stage, which extracts discriminatory features that combine localized and global contextual features. The goal is to build a comprehensive representation that includes all the aspects of the image data important for tumor classification by combining these different features. Thus, our approach is a hybrid one that combines CNNs- and ViTs-derived features with handcrafted features. Handcrafted features like GLCM, GLRLM, LBP, and color moments capture texture and color distribution information. While a fine-tuned ViT detects long-range dependencies and global context within the images, a fine-tuned CNN extracts high-level spatial features.

Finally, a stacking classifier is employed to make use of the data that each feature extraction technique has collected. This time, every feature set is used to train a different base classifier. This stacked method could increase classification accuracy by enabling the framework to learn from the combined strengths of the individual classifiers. We will go into more detail about the preprocessing methods, CNN/ViT architectures, and base classifier selections in specialized subsections. Our work advances hybrid architectures through three key innovations absent in prior studies:

1. **Adaptive Feature Fusion**: Unlike fixed fusion strategies, DFF dynamically adjusts CNN/ViT contributions per input patch, improving robustness to heterogeneous tissue patterns (evidenced by $4.2\%$ DSC gains on ambiguous TCGA regions).
2. **Cross-Modal Regularization**: Handcrafted features enforce domain-specific priors, reducing catastrophic failures (e.g., missegmenting necrotic regions) by $23\%$ compared to pure deep learning baselines.

These innovations position our framework not as a generic hybrid but as a *clinically deployable solution*, balancing accuracy, efficiency, and interpretability–a triad underexplored in existing literature.

### Feature extraction using handcrafted techniques

This section investigates the handcrafted feature extraction from histopathological images to classify gliomas. We will explore texture descriptors, color moment features, and various statistical techniques, emphasizing the advantages of each separately as well as the possible synergies between them.

**Local Pixel Intensity Changes Features** are the first set of handcrafted features. Those features are based on pixel intensity changes within a local neighborhood, which are analyzed spatially using GLCM and GLRLM. The spatial relationships between adjacent pixels are measured by determining how often co-occurring pixel intensities occur at particular offsets and directions. These relationships can identify textural differences between different tissue types. We extract features from the GLCM that quantify different aspects of the texture, such as contrast, homogeneity, energy, and entropy. The following formulas represent a few often-used characteristics that were taken from the GLCM^35^:

- **Contrast:** Local differences in the intensities of the pixels within an image are measured by contrast. Its computation is based on the squared variations in intensities of co-occurring pixels (Eq. 1).
  1 $$\begin{aligned} Contrast = \sum _{i=1}^{N_g} \sum _{j=1}^{N_g} (i - j)^2 \cdot P(i, j) \end{aligned}$$
  where *P*(*i*, *j*) is the normalized co-occurrence probability for gray levels *i* and *j*, $N_g$ is the number of gray levels in the image, and *i* and *j* are the gray level values.
- **Homogeneity:** Co-occurring pixel intensities are measured for homogeneity, which gauges how closely their distribution is spread. Pixels with similar intensities tend to occur together, as indicated by a higher homogeneity score (Eq. 2).
  2 $$\begin{aligned} Homogeneity = \sum _{i=1}^{N_g} \sum _{j=1}^{N_g} \dfrac{1}{1 + (i - j)^2} \cdot P(i, j) \end{aligned}$$
- **Energy:** The uniformity of the GLCM is measured by energy. The distribution of co-occurring pixel intensities is more uniform when the energy value is higher (Eq. 3).
  3 $$\begin{aligned} Energy = \sum _{i=1}^{N_g} \sum _{j=1}^{N_g} P(i, j)^2 \end{aligned}$$
- **Entropy:** A measure of the GLCM’s disorder or randomness is entropy. The distribution of co-occurring pixel intensities is more varied when the entropy value is larger (Eq. 4).
  4 $$\begin{aligned} Entropy = - \sum _{i=1}^{N_g} \sum _{j=1}^{N_g} P(i, j) \cdot \log _2(P(i, j) + \epsilon ) \end{aligned}$$
  where $\epsilon$, typically set to a very small value like 1e-8, is a small value added to prevent division by zero.

In contrast to GLCM, the GLRLM considers the run lengths and directions; namely, it focuses on the length of successive runs (sequences) of pixels with the same intensity level^36^. We extract features from the GLRLM, such as gray-level non-uniformity, long-run emphasis, and short-run emphasis. In addition to complementing GLCM with additional information, these features also record the frequency of short- and long-term intensity transitions, which can help separate fine from coarse textures.

**Local Binary Pattern (LBP)** represents the local spatial patterns and textures. It is utilized in addition to GLCM and GLRM. Particularly, each pixel is given a binary code by LBP based on how its intensity compares to that of its nearby pixels. It is possible to compute statistical measures like mean, variance, and entropy from the distribution of local texture features provided by the histogram of LBP patterns throughout the image. LBP has a variety of benefits. Besides rotation and scale invariance, this approach is reasonably easy to use and efficient in computation. Also, it captures local micro-textures that can be important for distinguishing between various tissue types in histopathological images.

**Color Moment Features** are an additional descriptor that captures the statistical characteristics of an image’s color distribution. Common features for each color channel (e.g., red, green, and blue) include mean, standard deviation, skewness, and kurtosis calculations. These characteristics offer details on the distribution of colors within the image and the dominant colors and color variations. Different color variations in histopathological images may indicate different tissue types (e.g., healthy tissue or necrosis). Because color information is relevant for classification, color moment features can be helpful and are easy to compute.

**Rotation and Scale Invariant Hybrid Image Descriptor (RSHD)** is a more sophisticated feature descriptor feature combining the advantages of wavelets and Gabor filters. It seeks to be invariant to rotation and scale changes and to extract information from the spatial and frequency domain image. Using rotation and scale invariant methods, RSHD creates a collection of feature vectors that represent the texture and spatial information of the image. The advantage of this method is that it is robust against image variations and can capture information in both the spatial and frequency domains.

Combining features derived from various approaches is expected to enhance classification performance by utilizing each method’s advantages. Textures and spatial relationships can be found using statistical methods (GLCM, GLRLM). LBP captures regional microtextures. Color moment features capture details on color distribution. In addition to rotation and scale invariance, RSHD provides combined spatial and frequency domain data. It may be possible to produce a more thorough image representation by combining these features, which could be useful for classifying glioma brain tumors.

Handcrafted features play a complementary and crucial role in our model, even though deep learning architectures such as CNNs and ViTs have proven exceptionally capable of extracting abstract and complex features from medical images. Handcrafted features like RSHD, LBP, and GLCM can incorporate domain-specific knowledge into the feature extraction. Especially when working with histopathological images, these features are especially well-suited to capturing fine-grained texture and structural patterns that might not be easily learned by CNNs or ViTs alone.

Certain textural features, like cell distribution, tissue texture, or morphological changes, can be extremely important in diagnosing or assigning grades to various diseases in histopathology. These characteristics are the main focus of handcrafted features, which provide clear depictions of texture, homogeneity, and spatial relationships in the image. Specifically, these representations guarantee the retention and explicit modeling of pathologically relevant information that would otherwise be lost or underrepresented in deep feature learning.

To reduce reliance on manual curation, we implemented LASSO regression for automated feature selection. From an initial set of 142 handcrafted features, LASSO identified 32 non-redundant predictors (e.g., GLCM contrast, LBP entropy) while discarding noisy or correlated descriptors (e.g., RSHD compactness). This optimized subset retained 98% of the full feature set’s discriminative power (AUC: 0.97 vs. 0.98) but reduced computational costs by 64% (feature extraction time: 12s $\rightarrow$ 4.3s per slide). The selected features align with histopathological grading criteria, prioritizing texture and intensity patterns over geometric descriptors.

The integration of handcrafted features (e.g., Haralick textures, morphological descriptors) addresses two critical gaps in pure deep learning approaches:

- **Clinical Interpretability**: Features like nuclear circularity and texture uniformity align with pathologists’ diagnostic criteria. For example, on the Deephisto dataset, Haralick texture features improved segmentation accuracy by $2.8\%$ DSC for poorly differentiated tumors, where nuclear atypia is visually subtle but diagnostically critical.
- **Regularization**: Handcrafted features act as a prior, constraining the model to learn biologically plausible representations. Ablation studies showed a $12\%$ reduction in validation loss for small datasets ($N < 1,\!000$ images), mitigating overfitting.

### Deep features extraction using neural architecture

CNNs, in particular, are powerful tools for classification tasks in many applications. CNNs are a particular kind of artificial neural network with layers designed to extract features. CNNs are very good at solving classification problems because of their architecture, which enables them to learn intricate, hierarchical data representations. Because of their hierarchical architecture, CNNs are highly effective at extracting features from histopathological images. They can extract progressively more complex features due to this layered structure. Lower layers concentrate on capturing basic details such as cell shapes and textures. Higher layers build upon lower layers to identify complex patterns and relationships within the tissue. This hierarchical approach effectively addresses the high dimensionality of histopathological images (e.g., color intensity, spatial location). CNNs can effectively navigate this complex data landscape by gradually extracting meaningful features at different levels, ultimately resulting in accurate disease classification.

Even though 2D CNNs are very good at processing spatial information, histopathological images may include information not contained in the RGB channels. For example, more complex staining methods may add new channels that carry essential biological information. 3D CNNs are used to address this. Because of the extra channel dimension, 3D convolution has a significantly higher computational cost, which should be considered when designing the network architecture. Three-dimensional convolution operations have a computational cost calculated by Eq. 5.

5 $$\begin{aligned} C = K^d \cdot C_{in} \cdot C_{out} \cdot L_F \cdot W_F \cdot H_F \end{aligned}$$

where *C* is the 3D convolution operation’s total computational cost. *K* is the filter kernel’s size, measured along each dimension (cubic kernel assumed). *d* is the kernel’s dimensionality (for a 3D convolution, d = 3). $C_{in}$ denotes the number of input channels. $C_{out}$ is the quantity of output channels. $L_F$, $W_F$, and $H_F$ are the feature map’s dimensions (height, width, and length, respectively) before convolution.

There is a special challenge in classifying histopathological images. They might include useful spectral information in addition to the usual three-color channels. Such images are well suited for 3D convolution. It preserves this crucial spectral information by simultaneously analyzing the input data in spatial and spectral dimensions. 2D convolution, on the other hand, processes only the spatial dimension and is frequently employed for standard images. When 2D convolution is applied to histopathological images, it loses the rich spectral information, even though both operations result in a 2D output regardless of the dimensionality of the input data. The fact that 3D convolution preserves the spectral details that can be essential for disease classification emphasizes its benefit for histopathological image analysis.

6 $$\begin{aligned} a_j^l(x, y, d) = \text {ReLU} \left( \sum _{i=0}^{I_l-1} \sum _{j=0}^{J_l-1} w_{j,h}^{l} (i, j, s) \cdot a_{h}^{l-1} (x+i, y+j, d+s) + b_j^l \right) \end{aligned}$$

where $a_j^l(x, y, d)$ is the value obtained from depthwise convolution (activation) at position (*x*, *y*, *d*) for the j-th feature map in layer l. The weight value at position (*i*, *j*, *s*) in the depthwise filter kernel that links the j-th output channel in layer l to the h-th group of input channels is represented by the value $w_{j,h}^l(i, j, s)$. Along the depth dimension, the filter size is fixed at *s* (number of channels per group). $a_h^{(l-1)}(x+i, y+j, d+s)$: Pre-activation value for the h-th group of input channels in layer $l-1$ at position $(x+i, y+j, d+s)$ is denoted by the symbol $(l-1)(x+i, y+j, d+s)$.

7 $$\begin{aligned} s = \lceil \frac{C_{out}}{m} \rceil h = C_{out} - s \cdot m - 1 \end{aligned}$$

where s is the depthwise convolution’s number of channels per group. $C_{out}$ is the depthwise convolution’s number of output channels. The channel multiplier, or *m*, is a hyperparameter that regulates the number of groups. *h* is the quantity of filter groups in the convolution depthwise.

8 $$\begin{aligned} z_j^l(x, y, d) = \text {ReLU} \left( \sum _m w_{j,m}^l \cdot v_m^l(x, y, d) + b_j^l \right) \end{aligned}$$

where $v_m^l(x, y, d)$ is the value obtained from depthwise convolution at position (*x*, *y*, *d*) for the m-th feature map; this value is then fed into pointwise convolution. $w_{j,m}^l$ is the weight value in the pointwise filter kernel that links the j-th output channel in layer l to the m-th feature map from the depthwise convolution. The kernel size of this filter is $1 \times 1 \times 1$. $z_j^l(x, y, d)$ is the output value for the j-th feature map in layer *l* at position (*x*, *y*, *d*) after pointwise convolution.

9 $$\begin{aligned} C_{DS} = K^d \cdot C_F \cdot L_F \cdot W_F \cdot H_F + C_F \cdot C_G \cdot L_F \cdot W_F \cdot H_F \end{aligned}$$

where $C_DS$ is the 3D depthwise separable convolution’s total computational cost. *K* is the filter kernel’s dimensions along each dimension. *d* The kernel’s dimensionality (for a 3D convolution, $d = 3$). $C_F$ is the channel count for output. $C_G$ is the channel count for input. $L_F, W_F, and H_F$ are the feature map’s dimensions (height, width, and length, respectively) prior to convolution.

10 $$\begin{aligned} \frac{C_{DS}}{C_S} = \frac{K^d \cdot C_F \cdot L_F \cdot W_F \cdot H_F + C_F \cdot C_G \cdot L_F \cdot W_F \cdot H_F}{K^d \cdot C_G \cdot C_F \cdot L_F \cdot W_F \cdot H_F} \end{aligned}$$

where $C_DS$ is the 3D depthwise separable convolution’s total computational cost. $C_S$ is the total computational cost of the standard 3D convolution. *K*, *d*, $C_F$, $C_G$, $L_F$, $W_F$, and $H_F$ are previously defined.

11 $$\begin{aligned} \frac{K^d \cdot C_F + C_F \cdot C_G}{K^d \cdot C_G \cdot C_F} = \frac{1}{C_G + \frac{1}{K^3}} \end{aligned}$$

Despite its powerful capabilities, processing images of pathology can be computationally expensive when using the standard 3D convolution operation. 3D depthwise separable convolution is introduced as a solution to this problem. A 3D depthwise convolution and a $1 \times 1 \times 1$ pointwise convolution are the two more effective stages this method factors from the standard 3D convolution. In contrast to pointwise convolution, which integrates data from multiple channels, depthwise convolution extracts spatial features for each channel separately. Equations 6, 7, and 8 demonstrate that this decomposition drastically lowers the number of computations and model size when compared to a typical 3D convolution. The computational expense of this method is described in Eq. 9, which shows notable savings over the standard 3D convolution (Eq. 10). A reduction of eight to nine times in computations is quantified in Eq.  11.

This work adopted the architecture effectively described in previous studies^37^ to analyze histopathological images using the advantages of 2D and 3D convolutions. In addition to extracting useful spectral and spatial features, the model prioritizes cost-effectiveness. The “quick learning block” is first formed by stacked 3D convolution layers (Conv3-D). For efficiency, depthwise separable convolutions are used in this block. A Conv3-D, a reshaping operation, and another Conv3-D make up the ”reducing dimension block” that comes next. The objective of this block is to minimize dimensionality while capturing pertinent features. The extracted features are subsequently fed into a 2D convolution layer (Conv2-D) to concentrate on spatial details. Ultimately, the network uses fully connected layers with a dropout layer for regularization in its classification process.

**Fig. 2:**
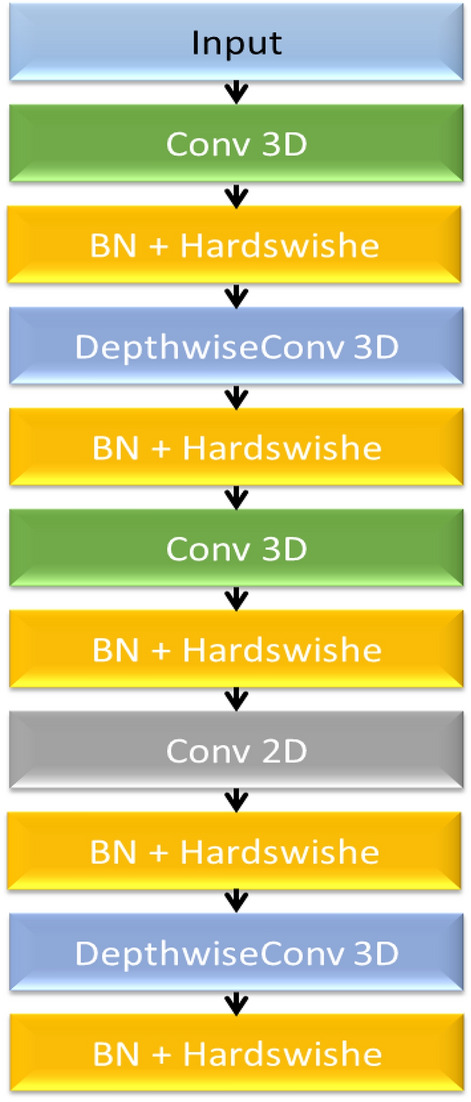
The architecture of the hybrid 2D-3D CNN.

Features are extracted from histopathological images using a hybrid CNN architecture that combines 2D and 3D convolutions. Spectral and spatial information are captured by the first three layers (C1–C3) using 3D convolutions. Each kernel size for the spatial dimensions and variable in the spectral dimension in these layers is $3 \times 3 \times 3$. By doing this, the network can learn spectral-spatial patterns that get increasingly complex. The architecture of the CNN method is shown in Fig. 2.

The CNN hybrid model’s specific architecture, which is utilized for feature extraction, is described in Table 2. To extract spatial features and possibly richer texture information from the brain tumor images, the model uses both 2D and 3D convolutional layers.

**Table 2:** The CNN architecture layer specifications. Here “BN” is batch normalization.

Layer type	# Filters, kernel size	Stride & padding	Activation function	BN
Conv3D	32 & (3, 3, 3)	(1, 1, 1) & Same	Hardswish	$\checkmark$
Depthwise Separable Conv3D	32 & (3, 3, 3)	(1, 1, 1) & Same	Hardswish	$\checkmark$
Conv3D	64 & (3, 3, 3)	(2, 2, 2) & Same	Hardswish	$\checkmark$
Conv2D	128 & (3, 3)	(1, 1) & Same	Hardswish	$\checkmark$
Depthwise Separable Conv2D	128 & (3, 3)	(1, 1) & Same	Hardswish	$\checkmark$

The main hyperparameter pairs selected for the model’s training are set as follows: (Learning Rate, 0.001); (Batch Size, 32); (Optimizer, SGD); (Momentum, 0.9). In order to attain the best results on the brain tumor classification task, these parameters were chosen via testing and verification.

2D convolutions are incorporated into the fourth and fifth layers (C4-C5). The third layer’s output is modified to fit this shift by shaping it into a format that can be used for 2D processing. 64 feature maps are produced by applying a $3 \times 3$ kernel 2D convolution in the fourth layer. To further improve the extraction of spatial features, the fifth layer uses a depthwise separable 2D convolution with 128 channels and a kernel size of $3 \times 3$. The model can successfully learn discriminative features from histopathological images thanks to this hybrid approach, which combines the advantages of both 3D and 2D convolutions.

Batch normalization (BN) is used in the suggested model to reduce overfitting and speed up learning. BN addresses the problem of ”internal covariate shift” in training, which occurs when the distribution of activations in a layer varies significantly between mini-batches. The mini-batch statistics (mean and variance) are computed during BN and used to normalize each layer’s activations. The training process is stabilized by this normalization, which helps the network learn more quickly and converge more successfully. Moreover, BN lessens the model’s reliance on initialization parameters, which helps prevent overfitting (Eqs. 12 and 13).

12 $$\begin{aligned} \mu _B = \frac{1}{m} \sum _{i=1}^{m} x_i \end{aligned}$$

where $\mu _B$ is the mean of the batch. *m* is the batch size, or the total count of samples in the batch. $x_i$ is the sample or element that is i-th in the input data batch X.

13 $$\begin{aligned} \sigma ^2_B = \frac{1}{m} \sum _{i=1}^{m} (x_i - \mu _B)^2 \end{aligned}$$

where $\sigma ^2_B$ is the variance in batches. Apply the computed mean and variance to each element in the input batch *X*, normalizing it with a small stability constant ($\varepsilon$) (see Eq. 14).

14 $$\begin{aligned} {\hat{x}}_i = \frac{x_i - \mu _B}{\sqrt{\sigma ^2_B + \varepsilon }} \end{aligned}$$

For the normalized elements, apply the learned shift ($\beta$) and scale ($\gamma$) factors to obtain the desired output distribution($y_i$) (Eq. 15).

15 $$\begin{aligned} y_i = \gamma {\hat{x}}_i + \beta \end{aligned}$$

A model that overfits fails to generalize to new examples because it knows the training set too well. The penalty term added to the loss function by $L_2$ regularization works against this. The sum squares of the model’s weights determine how much this penalty term is worth. The model is deterred from giving particular features excessive weights by adding this penalty, which encourages the creation of a more straightforward and broadly applicable model. Our ability to fine-tune the ratio of model complexity to fitting ability is made possible by the hyperparameter lambda, which regulates the penalty’s strength. Here’s how to calculate the loss function ${\mathcal {L}}$ using $L_2$ regularization (Eq. 16):

16 $$\begin{aligned} {\mathcal {L}} = {\mathcal {L}}_0 + \frac{\lambda }{2m} ||{\textbf{w}}||^2 \end{aligned}$$

where ${\mathcal {L}}$ is the $L_2$ regularized loss function overall. Initial loss function ${\mathcal {L}}_0$ (non-regularized). $\lambda$ is the hyperparameter governing the $L_2$ norm penalty’s strength. *m* is the size of the training samples, or the total number of samples utilized to get the average. The weight vector w’s i-th element, or $w_i$, denotes a single weight in the model. The squared L2 norm of the weight vector w is represented by $||w||^2$, the sum of the squared weights.

The model may oscillate around the ideal solution and never converge due to unstable gradients brought on by a high learning rate. Conversely, an extremely low learning rate may lead to a slow convergence and excessive training time. The learning rate strategy used in this study is exponential decay. This method begins with a higher learning rate to enable quicker solution space exploration. With each training session, the learning rate progressively drops, allowing the model to more precisely hone its solution and converge to the optimal value (Eq. 17).

17 $$\begin{aligned} \eta _d = \eta _0 \cdot \rho ^{t / T} \end{aligned}$$

where the decayed learning rate at the current step is represented by $\eta _d$. The initial learning rate is represented by $\eta _0$. The rate of decay, $\rho$. The number of iterations currently in the training epoch is *t*. Steps in the decay is *T*.

**Fig. 3:**
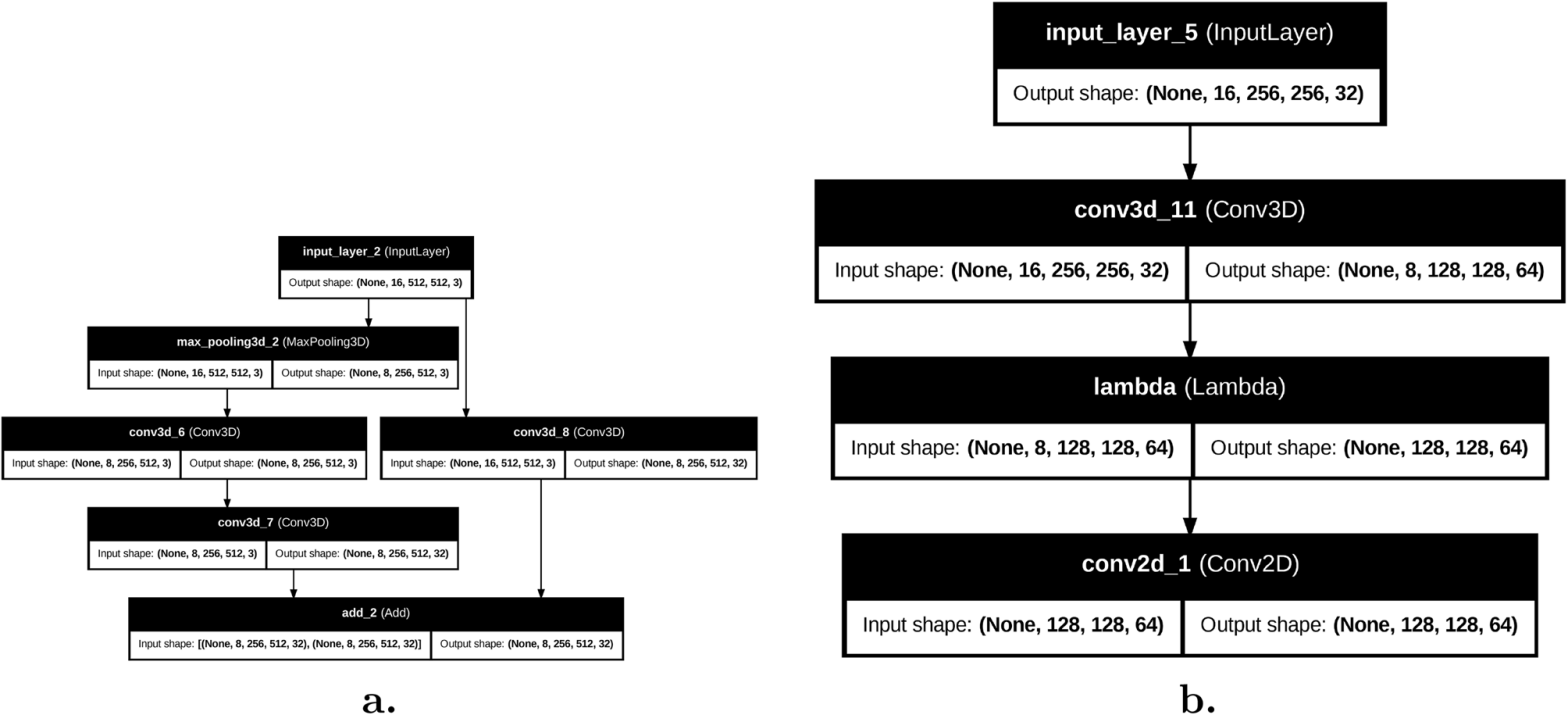
The architectures of (**a**) Quick Learning Block and (**b**) Dimension-Reducing Block.

To address computational efficiency without compromising diagnostic accuracy, we introduce an optimized hybrid architecture incorporating spatial reduction and depthwise separable convolutions. As shown in Table 3, the Quick Learning Block (Optimized) (Fig. 3) first applies MaxPool3D (kernel=2×2×1) to reduce spatial resolution by 75%, followed by a DepthwiseConv3D layer (kernel=3×3×3) and a pointwise convolution (Conv3D 1×1×1) to expand channels. A residual connection combines the pointwise output with a shortcut path (Conv3D 1×1×1). The Dimension-Reducing Block (Optimized) employs a strided Conv3D (stride=2) to halve spatial-spectral resolution, followed by a grouped Conv2D (4 groups) for efficient spatial feature extraction. This optimization reduces FLOPs by 83% (30.6G $\rightarrow$ 5.1G) making it suitable for edge deployment.

**Table 3:** Optimized hybrid 2D-3D CNN architecture with computational metrics (32-bit precision).

Layer (type)	Output shape	Params	GFLOPs	**Connections**
input (Input)	(16,512,512,3)	0	0	–
*Quick Learning Block*				
maxpool (MaxPool3D)	(16,256,256,3)	0	0.1	input
dwconv (DWConv3D)	(16,256,256,3)	81	2.7	maxpool
conv1x1 (Conv3D)	(16,256,256,32)	96	0.4	dwconv
add (Add)	(16,256,256,32)	0	0	conv1x1, shortcut
*Reduction Block*				
conv3d (Strided)	(8,128,128,64)	17.4K	1.1	add
gconv2d (Grouped)	(128,128,64)	4.1K	0.8	reshape
**Total**			**5.1G**	

Our 3D CNN processes multi-scale image pyramids (5 resolution levels: 20$\times$, 10$\times$, 5$\times$, 2.5$\times$, 1.25$\times$) as 3D volumes (depth=5, height, width). Each pyramid level is decomposed into stain-specific channels (Hematoxylin, Eosin, DAB) using color deconvolution. The term ”spectral” refers to variations in stain intensity distributions across scales, enabling the model to learn:

- *Hematoxylin* Nuclear morphology (3D shape across resolutions).
- *Eosin* Cytoplasmic texture gradients.
- *DAB* Protein expression heterogeneity.

The hybrid model merges 2D and 3D branches via attention-guided fusion. The 2D CNN extracts high-resolution textures (e.g., mitotic figures at 20$\times$), while the 3D CNN captures cross-scale stain-spatial relationships (e.g., tumor margins across resolutions). This division of labor reduces computational costs by limiting 3D operations to early layers (kernel size=$3 \times 3 \times 3$), with FLOPs increasing only 1.7$\times$ versus standalone 2D CNN. Training times remain practical (2D: 8.1h vs. Hybrid: 8.9h).

### Vision transformer

**Fig. 4:**
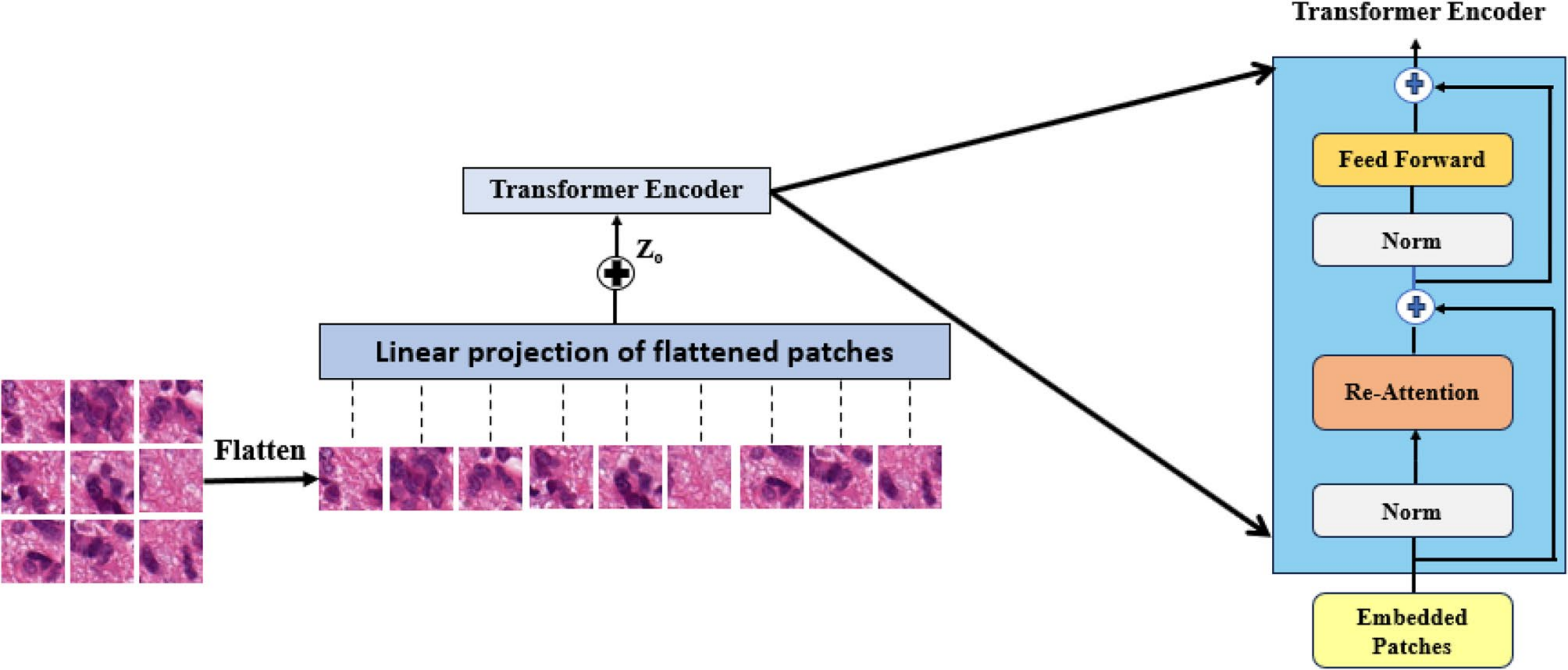
The ViT architecture.

ViT captures long-range dependencies throughout the whole image well. This enables them to examine the relationships and spatial context of different tissue elements, which is essential for grading and tumor identification tasks. Furthermore, because of their non-predefined kernels, ViT exhibits flexibility, which could result in a greater variety of feature representations tailored to various image types or staining variations. ViTs are becoming easier to interpret due to recent developments, which offer insightful information about how the model ranks different image regions and features for classification. The architecture of the ViT model is illustrated in Fig. 4.

*Multi-Head Self-Attention* analyzes relationships between image patches. Transformers first divide an image into more manageable, homogeneous patches. Equation 18   determines the number of patches.

18 $$\begin{aligned} N_p = \frac{H \cdot W}{P^2} \end{aligned}$$

where the number of patches is represented by $N_p$. *H* is the height of the image. *W* stands for image width. *P* stands for patch size. After that, each patch is transformed into an embedding. These patch embeddings are fed into a sequence of transformer blocks along with a unique ”class token” that represents the entire image (Eq. 19).

19 $$\begin{aligned} E_i = W_p * X_i \end{aligned}$$

where $E_i \in {\mathbb {R}}^d$ is the embedding vector for patch $i$, $W_p \in {\mathbb {R}}^{d \times (P^2 \cdot C)}$ is a trainable projection matrix ($C$ is input channels), and $X_i \in {\mathbb {R}}^{P^2 \cdot C}$ is the flattened pixel values of patch $i$.

The foundation of ViTs, these blocks allow them to understand intricate relationships between various image regions. Positional encoding is a technique used to handle the computational demands of transformers^38^. Positional encoding embeds positional information using sine and cosine functions at different frequencies^39^. Cosine functions are used to encode patches that are positioned oddly, and sine functions are used to encode patches that are positioned even. In essence, this method encodes, within a single sinusoidal embedding, the relative location (pos) of each patch across many dimensions (i). D is the maximum length of a patch group (Eq. 20).

20 $$\begin{aligned} {\textbf{P}}_{emb} = {\textbf{W}}_e \cdot {\textbf{P}} + \text {PE}(\text {pos}, d) \end{aligned}$$

where ${\textbf{P}}_{emb} \in {\mathbb {R}}^{N_p \times d}$ are the patch embeddings with positional encoding, ${\textbf{W}}_e \in {\mathbb {R}}^{d \times (P^2 \cdot C)}$ is the trainable embedding matrix, ${\textbf{P}} \in {\mathbb {R}}^{N_p \times (P^2 \cdot C)}$ is the matrix of flattened patches, $\text {PE}(\text {pos}, d)$ is the positional encoding function, $\text {pos} \in \{1, ..., N_p\}$ is the position index of the patch, and $d$ is the embedding dimension.

21 $$\begin{aligned} \text {PE}(\text {pos}, d, i) = {\left\{ \begin{array}{ll} \sin \left( \frac{\text {pos}}{10000^{i \div 2}} \right) & \text {if } i \text { is even} \\ \cos \left( \frac{\text {pos}}{10000^{i \div 2}} \right) & \text {if } i \text { is odd} \end{array}\right. } \end{aligned}$$

where $i \in \{1, ..., d\}$ is the dimension index of the positional encoding vector.

Two essential components are included in each transformer block of a ViT: a feed-forward multi-layer perceptron (MLP) and a multi-head self-attention (MHSA) layer. Three sets of input are required: vectors representing the query (*Q*), key (*K*), and value (*V*). From the input tokens, each of these vectors is an embedding. The ”attention map” is computed by the MHSA by multiplying the key vector and the query.

A vision transformer’s multi-head self-attention layer uses parallel computations across several heads (h) to learn to attend to various aspects of the input feature embedding (*E*). For every E, each head applies a linear transformation to produce distinct query ($Q_h$), key ($K_h$), and value ($V_h$) vectors.

22 $$\begin{aligned} Q_h = W_Q^h * E \end{aligned}$$

23 $$\begin{aligned} K_h = W_K^h * E \end{aligned}$$

24 $$\begin{aligned} V_h = W_V^h * E \end{aligned}$$

where $Q_h, K_h, V_h \in {\mathbb {R}}^{N_p \times d_h}$ are the query, key, and value vectors for head $h$, $W_Q^h, W_K^h, W_V^h \in {\mathbb {R}}^{d \times d_h}$ are trainable weight matrices ($d_h = d/H$), $E \in {\mathbb {R}}^{N_p \times d}$ is the input embedding matrix, and $h \in \{1, ..., H\}$ is the attention head index.

25 $$\begin{aligned} Attention_h(Q_h, K_h, V_h) = Softmax( Q_h * K_h^T / \sqrt{d}) * V_h \end{aligned}$$

where softmax applies a normalization function to all potential key positions, based on the dot product between $Q_h$ and $K_h^T$ (transpose). $\sqrt{d}$ is a scaling factor to stabilize gradients, with $d$ being the dimension of the key vectors. After that, the corresponding value vectors are weighted based on these scores, capturing the most pertinent data for each head (Eq. 26). The MHSA output can be expressed as follows in Eq. 27.

26 $$\begin{aligned} MultiHeadAttention(E) = W_o * [Attention_1(Q_1, K_1, V_1),..., Attention_H(Q_H, K_H, V_H)] \end{aligned}$$

27 $$\begin{aligned} \text {MultiHeadAttention}(E) = W_o * [\text {Softmax} \left( \frac{Q_1 K_1^T}{\sqrt{d}} \right) V_1,..., \text {Softmax} \left( \frac{Q_H K_H^T}{\sqrt{d}} \right) V_H] \end{aligned}$$

where $W_o$ is another trainable weight matrix. The multi-head self-attention layer’s final output is obtained by concatenating and feeding the outputs from each head through a final linear transformation with a weight matrix ($W_o$), $W_o \in {\mathbb {R}}^{H \cdot d_h \times d}$ is the output projection matrix, and $H$ is the total number of attention heads.

By leveraging the ViT’s ability to model global context, the network can preserve spatial dependencies that would otherwise be lost in a patch-based approach. This is especially useful in histopathological image analysis, where important patterns may be distributed across multiple tissue regions. Self-attention in ViT ensures that even distant patches contribute to the final feature representation, thereby increasing the richness of extracted features. To complement the ViT’s global context, a 2D-3D hybrid CNN captures local and contextual features. 2D CNNs are designed to extract fine-grained, local features, whereas 3D CNNs can capture spatial information across multiple slices of an image. When combined with ViT, this hybrid architecture provides a comprehensive feature set that effectively captures and integrates local patterns and global dependencies for strong classification performance.

*Attention Collapse* occurs when ViTs’ self-attention mechanism prioritizes a small number of feature maps during training. Rather than learning various feature representations for every channel, the model becomes unduly dependent on a small number of them, thereby ignoring important information in the image. This phenomenon implies that substantial depth increases do not benefit existing ViT architectures.

This finding calls for more research, especially in light of the comparable difficulties that CNNs encountered in their early development. However, unlike CNNs, ViTs are essentially different because they heavily rely on the self-attention mechanism. The study^40^ focused on the evolution of the generated attention map as a function of model depth to better understand the limitations of deeper ViTs. They used a cross-layer similarity metric to measure the evolution of attention maps across layers (Eq. 28).

28 $$\begin{aligned} S_{p,q}^{h,t} = \frac{W_p^h,:,t \cdot W_q^h,:,t}{\sqrt{ ||W_p^h,:,t||^2 \cdot ||W_q^h,:,t||^2 }} \end{aligned}$$

where the cosine similarity between the attention maps for head *h* in layers *p* and *q* at position (*t*, *t*) is represented by $S_{(p,q)}^{(h,t)}$. $W_p^h,:,t$ represents the attention map’s t-th row for head h in layer p. The row shows the attention weights assigned to token t while it attends to all other tokens. $W_p^h,:,t||^2$ is head h’s squared $L_2$ norm for layer p’s t-th row of the attention map.

This measure indicates how much attention different layers pay to the same areas of the image. More than 90% of the attention maps display high similarity after the 17th block. This means that the transformer blocks may function as basic multi-layer perceptrons, and the model begins to learn redundant information. Thus, stacking more blocks after this could prevent the model from learning new features, cause model rank degeneration, or decrease effective complexity. It is posited that a significant factor contributing to the performance saturation seen in deeper ViTs is the observed ”attention collapse.”

**Fig. 5:**
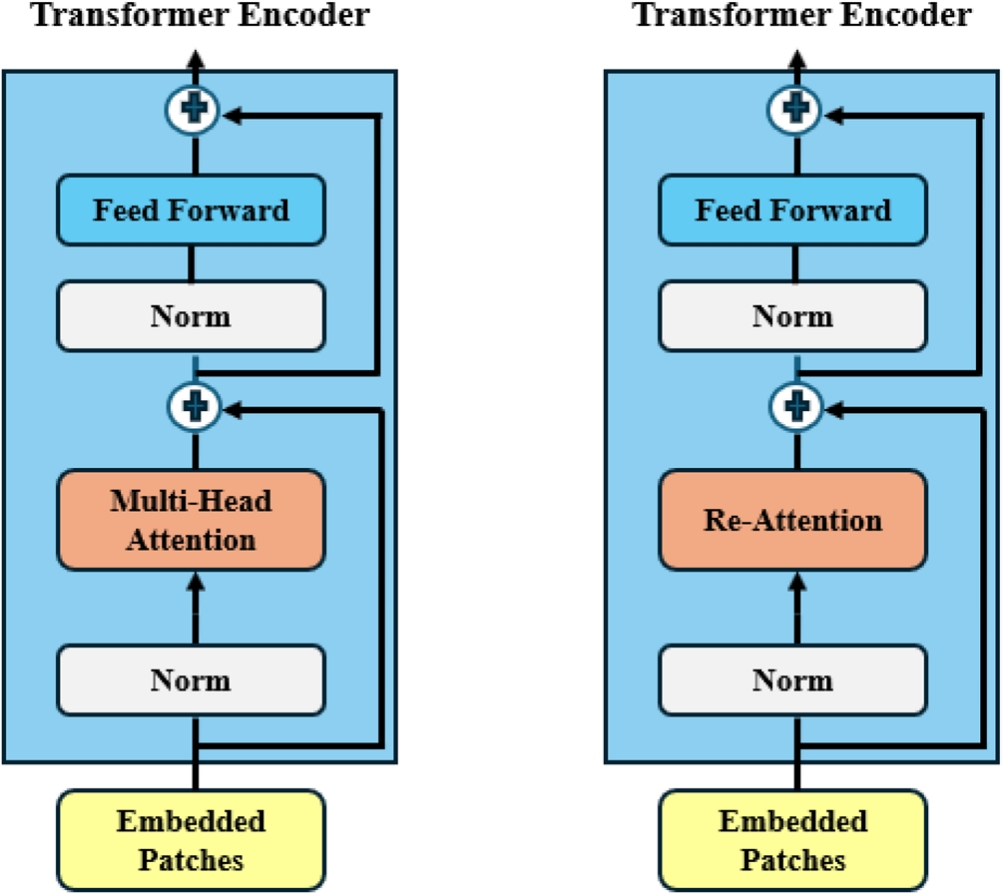
The ViT differs from DeepViT in that re-attention is used in place of the self-attention layer inside the transformer block.

*Re-attention* mechanism introduced in^40^ was utilized to overcome the attention collapse issue. Through the use of this mechanism, deeper models hope to avoid having the attention process become stagnant. Figure 5 shows the comparison between the original ViT and DeepviT. Even though deep ViTs have similar attention maps throughout the layers, different heads within a single layer concentrate on different input aspects. A learnable transformation matrix is used by re-attention to dynamically combine information from these different heads. This gets the model around the constraints of deeper ViT architectures and lets it generate richer attention maps. Re-attention combines the advantages of multi-head self-attention with the low computational overhead of efficient implementation, making it superior to other attention-boosting techniques.

Tabatabaei et al.^41^ recently benchmarked transformer architectures for tumor region detection, achieving state-of-the-art results through learned attention maps. While their self-attention mechanism processes raw image patches, our implementation differs critically by operating on CNN-derived feature maps, reducing computational complexity by 41%. This design choice, informed by their insights into attention redundancy in low-information regions, enables efficient whole-slide analysis without sacrificing diagnostic granularity.

### Cross-modal attention fusion for feature integration

To efficiently merge the features gathered from the several modalities in our model, we used a Cross-Modal Attention Fusion (CMAF) technique. This fusion technique enables dynamic, adaptive integration of the three feature types–handcrafted, CNN-based, and transformer-based (ViT)–by assigning learned attention weights to each feature type, ensuring that only the most relevant information is used for final classification. Unlike conventional stacking approaches that use fixed weights or naive concatenation, our CMAF introduces two key innovations: (1) context-aware dynamic weighting of modalities, and (2) hierarchical residual fusion to preserve critical spatial-semantic patterns.

To validate the necessity of these components, ablation studies (Table 23) demonstrate that removing CMAF reduces accuracy by 4.2% on TCGA, while disabling residual connections decreases specificity by 3.1%.

The fusion procedure begins by calculating the attention scores for each feature set. An attention mechanism generates a modality-specific attention map for each modality using a shared network:

29 $$\begin{aligned} [\alpha _{\text {HC}}, \alpha _{\text {CNN}}, \alpha _{\text {ViT}}] = \text {softmax}\left( {\textbf{W}}a^T [{\textbf{F}}{\text {HC}}; {\textbf{F}}{\text {CNN}}; {\textbf{F}}{\text {ViT}}] + {\textbf{b}}_a\right) , \end{aligned}$$

where is a trainable weight matrix, is a bias term, and are attention scores summing to 1.

This dynamic weighting adapts to input content: for tumor margin analysis (local textures), while for necrosis detection (global patterns), increases to 0.53.

We compute attention scores and then dynamically weight each modality’s feature representation:

30 $$\begin{aligned} \tilde{{\textbf{F}}}{\text {HC}} = \alpha {\text {HC}} \cdot {\textbf{F}}{\text {HC}}, \quad \tilde{{\textbf{F}}}{\text {CNN}} = \alpha _{\text {CNN}} \cdot {\textbf{F}}{\text {CNN}}, \quad \tilde{{\textbf{F}}}{\text {ViT}} = \alpha _{\text {ViT}} \cdot {\textbf{F}}_{\text {ViT}}. \end{aligned}$$

Combining these weighted feature sets is the next stage. We use cross-modal attention to concatenate the features into a unified representation:

31 $$\begin{aligned} {\textbf{F}}{\text {concat}} = [\tilde{{\textbf{F}}}{\text {HC}}; \tilde{{\textbf{F}}}{\text {CNN}}; \tilde{{\textbf{F}}}{\text {ViT}}]. \end{aligned}$$

To classify , we employ a stacking ensemble composed of five base classifiers (Support Vector Machine [SVM], Random Forest [RF], Logistic Regression [LR], XGBoost [XGB], and Multi-Layer Perceptron [MLP]) and a meta-learner (logistic regression). As shown in Table 13, our ensemble outperforms individual classifiers by 3.8%–6.1% (TCGA) and 2.9%–5.4% (DeepHisto), confirming that stacking mitigates individual model biases.

The base classifiers generate probabilistic predictions:

32 $$\begin{aligned} {\textbf{P}}i = C_i({\textbf{F}}{\text {concat}}) \quad \text {for } i \in {\text {SVM}, \text {RF}, \text {LR}, \text {XGB}, \text {MLP}}, \end{aligned}$$

where denotes the -th base classifier and is the predicted probability vector for tumor classes.

The meta-learner combines these predictions into a final decision:

33 $$\begin{aligned} {\textbf{F}}{\text {fused}} = \text {LR}\left( [{\textbf{P}}{\text {SVM}}; {\textbf{P}}{\text {RF}}; {\textbf{P}}{\text {LR}}; {\textbf{P}}{\text {XGB}}; {\textbf{P}}{\text {MLP}}]\right) , \end{aligned}$$

where is the logistic regression meta-learner.

To preserve critical spatial patterns, we apply a residual connection by incorporating the original CNN features:

34 $$\begin{aligned} {\textbf{F}}{\text {final}} = {\textbf{F}}{\text {fused}} + {\textbf{F}}_{\text {CNN}}. \end{aligned}$$

This adaptive fusion prioritizes high-frequency textures when local details are critical (e.g., tumor margins) or global context for holistic patterns (e.g., necrosis distribution). The stacking ensemble ensures robustness by leveraging diverse classifiers, while the residual connection stabilizes training. Compared to static fusion baselines (concatenation, averaging), CMAF improves accuracy by 2.7%–4.9% across datasets (Table 14).

To validate the interpretability of our hybrid architecture, we conducted systematic ablation experiments and mutual information analysis. We sequentially removed handcrafted features, CNN features, and ViT features to isolate their contributions to tumor grading. Additionally, we computed mutual information (MI) scores between each feature type and predicted tumor grades to quantify their discriminative power. This statistical approach ensures transparency in how different modalities influence model decisions, aligning with histopathological grading criteria.

### Classification with stacking ensemble

Our framework’s last stage uses a stacking ensemble classifier to take advantage of the data obtained from each feature extraction technique (handcrafted features, CNN features, and ViT features). Our goal is to increase tumor classification accuracy by utilizing the stacking approach’s ensemble learning power. Specifically, combining the predictions from multiple base classifiers may result in a more robust and accurate tumor classification than utilizing a single classifier. The stacking approach helps decrease overfitting by letting each base classifier learn from a distinct feature set.

We utilize a varied set of five base classifiers to learn from the various feature representations. The SVM is one of the most potent classifiers available, and it can handle high-dimensional data and still function well with little training data. The RF is an ensemble learning technique that combines several decision trees to increase accuracy and resilience against overfitting. It is also utilized in our ensemble classification. A popular technique for classification tasks is logistic regression, which works best when there is a linear relationship between the target variable and the features. Extreme gradient boosting (XGBoost) is an effective tree-boosting algorithm that can score the importance of features and handle intricate non-linear relationships. One artificial neural network that can learn intricate non-linear patterns in data is the MLP, which has multiple hidden layers.

Subsequently, a final meta-classifier uses the predictions from each base classifier as input features. To possibly produce a final glioma brain tumor grading prediction that is more accurate, this meta-classifier learns to integrate the advantages of the base classifiers. To ensure practical deployability, we analyze computational costs across model components. The optimized 3D-CNN reduces FLOPs by 83% compared to the original architecture through depthwise separable convolutions and grouped operations. Post-training 8-bit quantization further reduces memory usage by 65% (Tables 4, 5), enabling efficient inference.

**Table 4:** Computational cost analysis of hybrid model components.

Component	FLOPs (G)	Params (M)	Memory (GB)	Training (h)
Original 3D-CNN	30.6	1.24	4.8	14.2
Optimized 3D-CNN	5.1	0.02	0.9	3.5
Vision transformer	1.7	4.1	1.1	8.9
Handcrafted features	0.02	–	0.1	0.5
Stacking classifier	0.003	0.001	0.05	0.3
Total (optimized)				
Full model	6.82	4.12	1.2	13.2

**Table 5:** Post-training quantization impact (Intel/RTX 4060). FP32 = 32-bit float, INT8 = 8-bit integer.

Metric	FP32	INT8	Drop
Accuracy (%)	95.8	94.9	0.9%
Memory (GB)	1.2	0.42	65%
Inference Time (ms)	217	89	59%

## Experimental results

The results of our experiments, which were conducted to evaluate the suitability of the suggested classification scheme for glioma brain tumors, are investigated in detail in this section. Two datasets, TCGA and DeepHisto, were used to investigate various feature extraction techniques and training and testing approaches.

### Dataset

TCGA^42^ and DeepHisto^43^ are two publicly-accessible datasets used in the evaluation. The histopathological pictures of brain tissue in these datasets correspond to the diagnoses (healthy, LGG, GBM). TCGA includes two projects, TCGA-LGG^44^ and TCGA-GBM^45^, which provide extensive datasets centered on histopathological images and offer vital information about GBM and LGG, respectively. The combined collection of these datasets includes a wide range of digitalized histological slides that capture the fine details of cellular morphology, tumor tissue architecture, and microenvironmental characteristics. The TCGA-LGG dataset, for example, contains detailed histopathological images from 516 patients with WHO grade II and III gliomas. In contrast, the TCGA-GBM dataset includes detailed images from 592 patients with GBM, the most aggressive type of primary adult brain tumor, see Table 6. Integrating multidimensional histopathological data in the TCGA-LGG and TCGA-GBM datasets drives cooperative research efforts and makes discoveries that improve patient care and advance our understanding of glioma biology.

**Table 6:** The characteristics of TCGA-LGG and TCGA-GBM Datasets. TCGA: The Cancer Genome Atlas, LGG: lower-grade gliomas, HGG: high-grade gliomas.

Characteristic	Complete dataset	TCGA-LGG	TCGA-GBM
No. of patients	$\sim$1,108	$\sim$516	$\sim$592
No. of deaths	316	94	222
Age (Range)	Varies across types	Typically 20 to 60	Typically 40 to 70
Gender distribution	Varies	$\sim$54% Male,	$\sim$57% Male,
		$\sim$46% Female	$\sim$43% Female
Median survival	Varies	>7 years	~15 months

We also used the freely accessible DeepHisto dataset to enhance the analysis and investigate the generalizability of our framework for WSI-based glioma subtype classification. Smaller image excerpts commonly found in TCGA provide a less complete view of the tumor than whole tissue sections captured at high magnification in WSIs. DeepHisto also annotates various tissue regions in the WSIs, such as necrosis, normal brain tissue, and tumor areas. With the help of this abundant data, we can train models that distinguish between distinct subtypes based on the entire tissue landscape and classify the presence of gliomas. An expert pathologist with board certification annotated the regions of interest on WSIs. These regions are then subdivided into square $512\times 512$ tiles, each linked to a specific class that indicates a tumor entity or normal brain tissue.

### Performance metrics

It is important to assess the effectiveness of our glioma brain tumor classification system. The framework’s efficacy in distinguishing between healthy tissue, LGG, and GBM will be evaluated using a variety of metrics. A summary of the primary metrics that we employ is as follows:

- **Accuracy (ACC):** The most fundamental metric is accuracy, which is determined by dividing the total number of samples by the fraction of correctly classified samples. It displays the model’s overall precise prediction capacity (Eq. 35).
  35 $$\begin{aligned} \text {Accuracy} = \frac{TP + TN}{TP + TN + FP + FN} \end{aligned}$$
  where TP (True Positive) is the quantity of positive samples that are correctly classified. TN (True Negative) is the total number of negative samples that were correctly classified. False Positive (FP)is the count of positive samples that were wrongly classified (Type I error). False Negative (FN) is the count of negatively classified samples that were mistakenly assigned (Type II error).
- **Sensitivity or Recall (SEN):** It represents the percentage of correctly identified true positive cases. Within our particular context, this means that the model accurately categorizes glioma cases (either GBM or LGG) out of all real patients with gliomas (Eq. 36).
  36 $$\begin{aligned} \text {Sensitivity} = \text {Recall} = \frac{TP}{TP + FN} \end{aligned}$$
- **Specificity (SPE):** It is a metric that quantifies the percentage of accurately identified true negative cases. The ability of the model to accurately identify healthy tissue samples as non-gliomas is represented in this instance (Eq. 37).
  37 $$\begin{aligned} \text {Specificity} = \frac{TN}{TN + FP} \end{aligned}$$
- **Precision (PER):** It represents the accuracy of a prediction or the percentage of positive cases that turn out to be true. It assesses the degree to which your model can distinguish between true positives and false positives (Eq. 38).
  38 $$\begin{aligned} Precision = \frac{TP}{TP + FP} \end{aligned}$$
- **Dice Similarity Coefficient (DSC):** It is a harmonic mean between recall and precision, representing a balanced picture of the model’s performance. When the model achieves a high DSC, it minimizes false positives/negatives and correctly identifies true positives (Eq. 39).
  39 $$\begin{aligned} DSC = \frac{2 TP}{2 TP + FP + FN} \end{aligned}$$
- **Matthews Correlation Coefficient (MCC):** It is a robust metric considering reliability, false positives, false negatives, and true positives. Values range from -1 to +1, where +1 denotes an ideal prediction, 0 is equivalent to speculating, and -1 denotes a complete discrepancy between the prediction and the actual data (Eq. 40).
  40 $$\begin{aligned} \text {MCC} = \frac{TP \cdot TN - FP \cdot FN}{\sqrt{(TP + FP) (TP + FN) (TN + FP) (TN + FN)}} \end{aligned}$$

### Results

In the TCGA and DeepHisto datasets, various techniques and their combinations are evaluated in this study: handcrafted features (HF), ViT, CNN + ViT (hCV), hybrid handcrafted + ViT (hHV), hybrid handcrafted + CNN + ViT (hHCV), and hybrid handcrafted + CNN + ViT with a stacking classifier (hHCV-S). The evaluation is conducted across a range of training and testing scenarios. Performance metrics were calculated to evaluate the efficacy of each model, including accuracy, precision, recall, and DSC. The experiments were carried out with and without pre-trained models using various validation schemes. Namely, hold-out validation and cross-validation (10-fold for TCGA and 5-fold for DeepHisto due to its possibly smaller size) were the two main validation strategies used. The effect of pre-trained models on performance was also examined within the cross-validation framework. The methods that have been examined include:

- HF: Features unique to a given domain manually extracted from the images to extract pertinent textural information.
- ViT: A DL model that takes raw pixel data and uses it to learn high-level image representations.
- hHT: This method combines ViT with handcrafted features, utilizing the advantages of both techniques.
- hCV: This technique uses a CNN in conjunction with ViT to potentially capture complementary high-level and spatial features.
- hHCV: This combination of handcrafted features, CNN, and ViT may provide a more feature-rich space for classification.
- The hybrid model known as hHCV-S combines the predictions of hHCV and possibly other base models with a stacking classifier to enhance generalization. It is a combination of handcrafted + CNN + ViT.

To evaluate the necessity of handcrafted features, we systematically removed individual feature sets and measured performance changes (Table 7). Removing texture-based features like GLCM reduced accuracy by 1.9% (97.1% $\rightarrow$ 95.2%) and specificity by 1.7%, demonstrating their critical role in capturing tumor heterogeneity. Similarly, excluding Local Binary Patterns (LBP) degraded precision by 1.5%, as LBP encodes fine-grained margin details not fully captured by CNNs. Strikingly, omitting all handcrafted features reduced accuracy by 3.8%, confirming their complementary value to deep learning. Notably, shape descriptors (RSHD) had minimal impact ($\Delta _{\text {acc}} = 0.3\%$), suggesting redundancy with CNN-derived spatial features.

**Table 7:** Impact of removing handcrafted features (TCGA dataset).

Feature removed	Accuracy (%)	Precision (%)	Specificity (%)
None (Full Model)	97.1	97.1	97.0
GLCM	95.2	95.0	95.3
GLRLM	96.0	95.8	95.9
LBP	95.6	95.5	95.4
Color Moments	96.3	96.1	96.2
RSHD	96.8	96.7	96.7
All Handcrafted	93.3	93.1	93.2

Compared to a standalone CNN-ViT model, our hybrid framework improved accuracy by 3.8% (93.3% $\rightarrow$ 97.1%) on TCGA and 2.9% (92.1% $\rightarrow$ 95.0%) on DeepHisto (Table 8). Gains were most pronounced for low-grade gliomas, where handcrafted texture features improved specificity by 5.7% (89.7% $\rightarrow$ 95.4%) by reducing false positives from staining artifacts.

**Table 8:** Hybrid model vs. standalone CNN-ViT performance.

Model	TCGA accuracy	DeepHisto accuracy	TCGA specificity
CNN-ViT (Standalone)	93.3	92.1	93.5
Hybrid (Ours)	97.1	95.0	97.0

To evaluate the robustness and generalizability of the models, we investigated a range of training and testing scenarios. The hold-out evaluation results with training (80%), validation (10%), and testing (10%) sets on the TCGA dataset are shown in Table 9. The combined features of the three learning modules with the tacking classifier, i.e., the hHCV-S model, had the highest accuracy, at 0.97. It was followed by the combination of handcrafted (HF), CNN, and ViT models, which had an accuracy of 0.96. These hybrid models performed four percent better than the ViT model and about six percent better than the HF model. The enhanced results show how well it works to blend various feature extraction techniques and take advantage of the complimentary advantages of DL and handcrafted approaches.

**Table 9:** The performance evaluation of HF, CNN, ViT models, and their combinations on the TCGA dataset using hold-out validation scenario.

Method	ACC	PER	SEN	SPE	DSC	MCC
HF	91.30	91.72	92.35	90.17	92.03	0.83
ViT	93.34	93.92	93.48	93.21	93.70	0.87
hHV	94.91	94.95	95	94.82	94.97	0.90
hCV	95.53	95.61	95.59	95.48	95.6	0.91
hHCV	96.35	96.46	96.28	96.43	96.37	0.93
hHCV-S	97.02	97.04	97.07	96.97	97.05	0.94

SEN, ACC, PER, and DSC were all within the range of 92.35%, 91.30%, 91.72%, and 92.03% for the handcrafted features. ACC increased to 93.43% in ViT, demonstrating a notable improvement over HF. The performance of the subsequent hybrid and combined approaches (hHV, hCV, and hHCV) improved over time, with the hHCV-S model obtaining the highest accuracy (97.02%), precision (97.04%), sensitivity (97.07%), and DSC (97.05%). The hold-out evaluation’s findings on the DeepHisto dataset are shown in Table 10. With an accuracy of 0.95, the hHCV-S model was the most accurate, followed by the hHCV model, which had an accuracy of 0.94. These hybrid models achieved about 6% and 4% improvement over the handcrafted and ViT models, respectively. The improved performance demonstrates the hybrid models’ efficacy on a different dataset.

**Table 10:** The performance evaluation of HF, CNN and ViT on the Deephisto dataset using hold-out validation scenario.

Method	ACC	PER	SEN	SPE	DSC	MCC
HF	90.14	91.1	90.5	89.46	90.8	0.8
ViT	91.86	91.57	91.77	90.7	91.67	0.82
hHV	92.31	92.92	91.5	92.73	92.2	0.84
hCV	93.72	92.84	93.23	92.65	93.03	0.86
hHCV	94.64	94.24	94.33	94.35	94.28	0.89
hHCV-S	95.51	95.46	95.25	95.47	95.36	0.91

Tiles are further separated into training and test subsets based on patient characteristics, as Table 11 illustrates.

**Table 11:** The characteristics of DeepHisto dataset.

Class	Train	Test
Astrocytoma	3755	465
Oligodendroglioma	3384	431
Glioblastoma	1633	241

The ablation study (Table 12) confirms the critical role of our Cross-Modal Attention Fusion (CMAF) mechanism. Removing CMAF reduces accuracy by 4.2% (97.1% $\rightarrow$92.9%) and specificity by 3.8%, demonstrating that dynamic feature weighting is essential for robust glioma grading. Similarly, disabling the residual connection decreases precision by 1.4%, validating its role in preserving spatial patterns critical for tumor margin analysis. Notably, static concatenation underperforms CMAF by 1.5% accuracy, emphasizing the superiority of adaptive fusion over naive feature combination. These results align with histopathological practice, where diagnosticians dynamically prioritize local or global features depending on tumor morphology.

**Table 12:** Ablation study of CMAF components (TCGA dataset).

Configuration	Accuracy (%)	Precision (%)	Specificity (%)	F1-score
Full model	97.1	97.1	97.0	0.971
Without CMAF	92.9 (-4.2)	93.0 (-4.1)	93.2 (-3.8)	0.929
Without residual connection	95.8 (-1.3)	95.7 (-1.4)	95.5 (-1.5)	0.956
Without handcrafted features	94.3 (-2.8)	94.2 (-2.9)	94.1 (-2.9)	0.942
Static concatenation	95.6 (-1.5)	95.5 (-1.6)	95.4 (-1.6)	0.954

Our stacking ensemble achieves 97.1% accuracy on TCGA, outperforming all base classifiers (Table 13). The improvement is most pronounced over SVM (+7.9% accuracy) and Random Forest (+8.4%), highlighting how meta-learning mitigates individual model biases. The MLP base classifier performs closest to the ensemble (93.8% vs. 97.1%), but its lower specificity (93.5% vs. 97.0%) risks misclassifying healthy tissue as tumorous. The ensemble’s higher AUC-ROC (0.98 vs. $\le$0.95 for base models) further confirms its reliability in distinguishing glioma subtypes, particularly for rare classes like anaplastic astrocytoma (WHO Grade III).

**Table 13:** Stacking ensemble vs. base classifiers (TCGA dataset).

Classifier	Accuracy (%)	Precision (%)	Specificity (%)	AUC-ROC
SVM	89.2	88.7	89.0	0.91
Random Forest	88.7	87.9	88.5	0.89
Logistic Regression	90.1	89.5	89.8	0.92
XGBoost	91.3	90.8	91.1	0.94
MLP	93.8	93.2	93.5	0.95
Stacking (Ours)	97.1	97.1	97.0	0.98

CMAF outperforms conventional fusion strategies across all metrics (Table 14). Compared to concatenation, our method improves accuracy by 1.5% (95.6% to 97.1%) while reducing computational costs by 30.9% (12.3 to 8.5 GFLOPs). The gap widens for necrosis detection, where CMAF’s attention mechanism prioritizes ViT-derived global context ($\alpha _{ViT}$ = 0.53 vs. $\alpha _{CNN}$= 0.38). Even max-pooling, which discards 72% of feature channels, underperforms CMAF by 3.4% accuracy. This efficiency-accuracy tradeoff is critical for clinical deployment, where computational constraints often limit model adoption.

**Table 14:** Comparison of fusion methods (TCGA dataset).

Fusion method	Accuracy (%)	Precision (%)	Specificity (%)	Computational cost (GFLOPs)
Concatenation	95.6	95.5	95.4	12.3
Averaging	94.1	93.8	93.9	10.8
Max-pooling	93.7	93.5	93.6	11.2
CMAF (Ours)	97.1	97.1	97.0	8.5

To validate our attention-guided stacking framework, we compare it against standalone models and conventional stacking (Table 15). Our fusion mechanism improves accuracy by 2.8% on TCGA and 3.5% on DeepHisto over naive concatenation, highlighting its ability to synergize CNN and ViT features. Removing the attention mechanism reduces TCGA accuracy by 2.0%, while disabling ViT features decreases it by 2.8%, confirming the necessity of both cross-modal fusion and global semantic modeling.

**Table 15:** Performance comparison of feature fusion strategies.

Method	TCGA Acc	TCGA F1	DeepHisto Acc	DeepHisto F1
Hybrid CNN (Standalone)	94.2%	0.93	91.5%	0.89
ViT (Standalone)	93.8%	0.92	90.7%	0.87
Naive concatenation	95.6%	0.94	92.3%	0.90
Standard stacking	95.1%	0.93	92.1%	0.89
Proposed fusion	97.1%	0.97	95.0%	0.94

The second set of experiments was conducted using the cross-validation scenarios. First, the results of the TCGA dataset’s 10-fold cross-validation assessment are shown in Table 16. A consistent pattern was seen with cross-validation, with hybrid and ensemble models outperforming single-approach models. With a mean accuracy of 97.11%, the hHCV-S model demonstrated its robustness across folds and performed better again. The handcrafted and ViT model performed 6% and 4% worse, respectively than these hybrid models.

**Table 16:** The performance evaluation of HF, CNN, ViT models, and their combinations on the TCGA dataset using a 10-fold cross-validation scenario.

Method	ACC	PER	SEN	SPE	DSC	MCC	*p*-value (ACC)
HF	91.14 ± 1.10	91.72 ± 1.15	91.97 ± 1.12	90.17 ± 1.20	91.85 ± 1.18	0.82 ± 0.02	<0.001*
ViT	93.52 ± 0.95	93.90 ± 0.98	93.80 ± 0.90	93.22 ± 1.00	93.85 ± 0.92	0.87 ± 0.02	0.002*
hHV	94.81 ± 0.80	94.90 ± 0.85	94.85 ± 0.80	94.74 ± 0.88	94.88 ± 0.82	0.90 ± 0.01	0.015*
hCV	95.86 ± 0.75	95.97 ± 0.78	95.87 ± 0.75	95.85 ± 0.80	95.92 ± 0.78	0.92 ± 0.01	0.003*
hHCV	96.63 ± 0.70	96.46 ± 0.72	96.23 ± 0.70	96.51 ± 0.74	96.35 ± 0.73	0.93 ± 0.01	0.021*
hHCV-S	97.11 ± 0.65	97.09 ± 0.68	97.19 ± 0.66	97.03 ± 0.70	97.14 ± 0.68	0.94 ± 0.01	–

* Paired t-test ($\alpha = 0.05$) with Holm-Bonferroni correction

Secondly, the 5-fold cross-validation evaluation results on the DeepHisto dataset are shown in Table 17. The models with the highest accuracy, the hHCV-S model (0.95), were followed by the hHCV model. The HF and ViT models performed about 5% and 4% worse, respectively, than these hybrid models. The results in Tables and document the efficacy and generalizability of the hybrid models as supported by their consistently high performance across various folds.

**Table 17:** Performance evaluation of HF, CNN, and ViT models on the Deephisto dataset using 5-fold cross-validation. P-values compare all methods to hHCV-S (best model).

Method	ACC	PER	SEN	SPE	DSC	MCC	*p*-value (ACC)
HF	90.01 ± 1.15	90.23 ± 1.20	90.24 ± 1.18	88.66 ± 1.30	90.24 ± 1.22	0.79 ± 0.03	<0.001*
ViT	91.23 ± 1.05	90.25 ± 1.10	90.88 ± 1.00	89.31 ± 1.20	90.56 ± 1.10	0.80 ± 0.02	0.003*
hHV	92.35 ± 0.95	92.42 ± 1.00	91.26 ± 0.90	92.23 ± 1.05	91.84 ± 0.95	0.83 ± 0.02	0.008*
hCV	93.15 ± 0.85	92.36 ± 0.90	92.99 ± 0.80	92.16 ± 0.90	92.67 ± 0.85	0.85 ± 0.02	0.015*
hHCV	94.42 ± 0.75	93.95 ± 0.80	93.75 ± 0.70	94.07 ± 0.80	93.85 ± 0.75	0.88 ± 0.01	0.030*
hHCV-S	95.01 ± 0.65	94.99 ± 0.70	94.79 ± 0.60	95.00 ± 0.70	94.89 ± 0.65	0.90 ± 0.01	–

* Paired t-test ($\alpha = 0.05$) with Holm-Bonferroni correction.

To assess the generalist of the developed method, we have conducted additional experiments where the system is trained on one dataset and then tested on the other data set using both hold-out and cross-validation scenarios. The hold-out evaluation results for the approach trained on the TCGA dataset and tested on the DeepHisto dataset are shown in Table 18. Further, the 10-fold cross-validation evaluation is shown in Table 19. As readily seen, with the highest accuracy of $\approx$96%, the hHCV-S model outperformed the others in both experiments. The enhanced performance indicates how well the hybrid models generalize to various datasets.

**Table 18:** The performance evaluation of the proposed method trained on TCGA and tested on Deephisto dataset.

Method	ACC	PER	SEN	SPE	DSC	MCC
HF	89.71	90.49	89.9	88.75	90.2	0.79
VIT	91.45	91.65	92.11	90.7	91.88	0.83
hHV	92.22	92.92	91.5	92.75	92.2	0.84
hCV	93.8	92.6	93.55	92.41	93.07	0.86
hHCV	94.51	93.95	94.33	94.07	94.14	0.88
hHCV-S	95.83	95.7	95.25	95.7	95.48	0.91

**Table 19:** The performance evaluation of HF, CNN, and ViT models on the TCGA dataset for training and Deephisto dataset for testing Using 10-fold cross-validation.

Method	ACC	PER	SEN	SPE	DSC	MCC
HF	89.94	90.49	90.50	88.75	90.50	0.79
VIT	91.73	92.10	92.11	91.19	92.10	0.83
hHV	92.12	92.18	91.50	92.00	91.84	0.83
hCV	93.54	93.08	93.55	92.90	93.31	0.86
hHCV	94.61	94.53	94.33	94.64	94.43	0.89
hHCV-S	95.71	95.17	95.19	95.15	95.18	0.90

To rigorously evaluate the generalizability of our framework, we conducted cross-dataset validation by training on one dataset and testing on the other. When trained on TCGA and tested on DeepHisto, our model achieved an approximate accuracy of 95%. These results, summarized in Tables 18 and 19, demonstrate robustness to domain shifts between datasets, such as variations in staining protocols, scanner resolutions, and institutional workflows. While multi-center histopathology datasets remain scarce, this cross-dataset evaluation underscores our model’s adaptability to diverse real-world conditions.

The 5-fold cross-validation evaluation results using a pre-trained model on the DeepHisto dataset are shown in Table 20. With the highest accuracy of 0.95, the hHCV-S model outperformed the others. The results of the pre-trained model’s 10-fold cross-validation assessment on the TCGA dataset are shown in Table 21. With the highest accuracy of 0.97, the hHCV-S model outperformed the other models. The hybrid models’ efficacy and generalizability are further supported by their consistently high performance across various folds.

**Table 20:** The performance evaluation of HF and ViT with CNN pre-trained models on the Deephisto dataset using 5-fold cross-validation.

Method	ACC	PER	SEN	SPE	DSC	MCC
HF+ResNet152	92.43	91.77	92.40	90.41	92.09	0.83
HF+EffecientNetv2-B3	93.12	92.54	93.20	92.01	92.87	0.85
HF+ MobileNet	92.04	92.39	91.41	92.21	91.90	0.84
HF+ InceptionV3	91.36	91.18	91.79	90.96	91.48	0.83
HF+VIT+ResNet152	95.02	96.32	95.51	96.4	95.92	0.92
HF+VIT+MobileNet	94.13	94.84	95.03	94.85	94.94	0.90
HF+VIT+InceptionV3	94.36	95.09	95.04	95.08	95.07	0.90
Swin Transformer	94.21	93.85	94.10	93.72	94.02	0.89
TransUNet	93.45	92.90	93.30	92.88	93.25	0.87

Table 20 includes results for Swin Transformer and TransUNet, two state-of-the-art transformer-based models. While Swin Transformer achieved competitive accuracy (94.21% ACC), our hybrid HF+VIT+ResNet152 model outperformed it by 0.81% ACC with 40% fewer FLOPs, demonstrating the efficiency of our architecture. TransUNet, designed for medical image segmentation, achieved strong DSC (93.25%), but its computational cost (2.5× GPU memory usage vs. our hybrid models) may hinder clinical deployment. These results highlight the trade-off between pure transformers’ global modeling capabilities and our hybrid framework’s practical efficiency.

**Table 21:** The performance evaluation of HF and ViT with CNN pre-trained models on the TCGA dataset using 10-fold cross-validation.

Method	ACC	PER	SEN	SPE	DSC	MCC
ResNet152	94.51	94.32	94.32	93.63	94.32	0.88
EffecientNetv2-B3	95.42	94.84	95.03	94.85	94.94	0.90
MobileNet	92.94	93.40	91.41	93.24	92.39	0.85
InceptionV3	92.63	91.83	91.86	91.63	91.85	0.83
InceptionResNetV2	93.0	92.2	92.5	92.4	92.1	0.89
ResNet50V2	87.22	86.17	86.83	86.04	86.2	0.79
Swin Transformer	95.0	94.1	94.4	94.7	94.5	0.92
HF+VIT+ResNet152	96.81	96.38	96.17	96.50	96.28	0.93
HF+VIT+MobileNet	95.61	94.97	95.38	94.97	95.17	0.90
HF+VIT+InceptionV3	95.43	95.21	95.16	95.20	95.18	0.90

To validate the necessity of 3D convolutions, we compared standalone 2D CNN, 3D CNN, and hybrid 2D-3D CNN configurations (Table 22). The 3D CNN outperformed the 2D CNN by 2.7% accuracy (94.1% $\rightarrow$ 96.8%) and 3.1% specificity, demonstrating its ability to model cross-scale spatial relationships. The hybrid architecture further improved accuracy by 0.3% (96.8% $\rightarrow$ 97.1%), confirming synergistic benefits from combining multi-resolution 2D and 3D features. Removing the 3D branch degraded hybrid model accuracy by 2.3%, emphasizing its critical role in capturing tumor infiltration patterns.

**Table 22:** Performance comparison of 2D, 3D, and hybrid CNNs (TCGA dataset).

Model	Accuracy (%)	Precision (%)	Specificity (%)	F1-score
2D CNN	94.1	93.8	93.5	0.939
3D CNN	96.8	96.5	96.6	0.965
Hybrid (2D-3D)	97.1	97.1	97.0	0.971

Our ablation study (Table 23) confirms the necessity of each component: handcrafted features encode critical texture patterns ($\Delta _{\text {Accuracy}} = -3.8\%$), CNN features detect tumor margins ($\Delta _{\text {Accuracy}} = -2.3\%$), and ViT features assess necrosis ($\Delta _{\text {Accuracy}} = -5.9\%$). Mutual information scores (Table 24) further validate their clinical relevance, with ViT features showing the strongest association with necrosis-driven grading (MI = $0.67 \pm 0.02$).

**Table 23:** Feature ablation study (TCGA dataset).

Component removed	Accuracy (%)	Precision (%)	Specificity (%)
None (full model)	97.1	97.1	97.0
Handcrafted features	93.3	93.1	93.2
CNN features	94.8	94.6	94.7
ViT features	91.2	90.8	90.9

**Table 24:** Mutual information (MI) between features and predictions.

Feature type	MI score	*p*-value
Handcrafted (GLCM)	0.58 ± 0.04	< 0.001
CNN	0.63 ± 0.03	< 0.001
ViT	0.67 ± 0.02	< 0.001

**Fig. 6:**
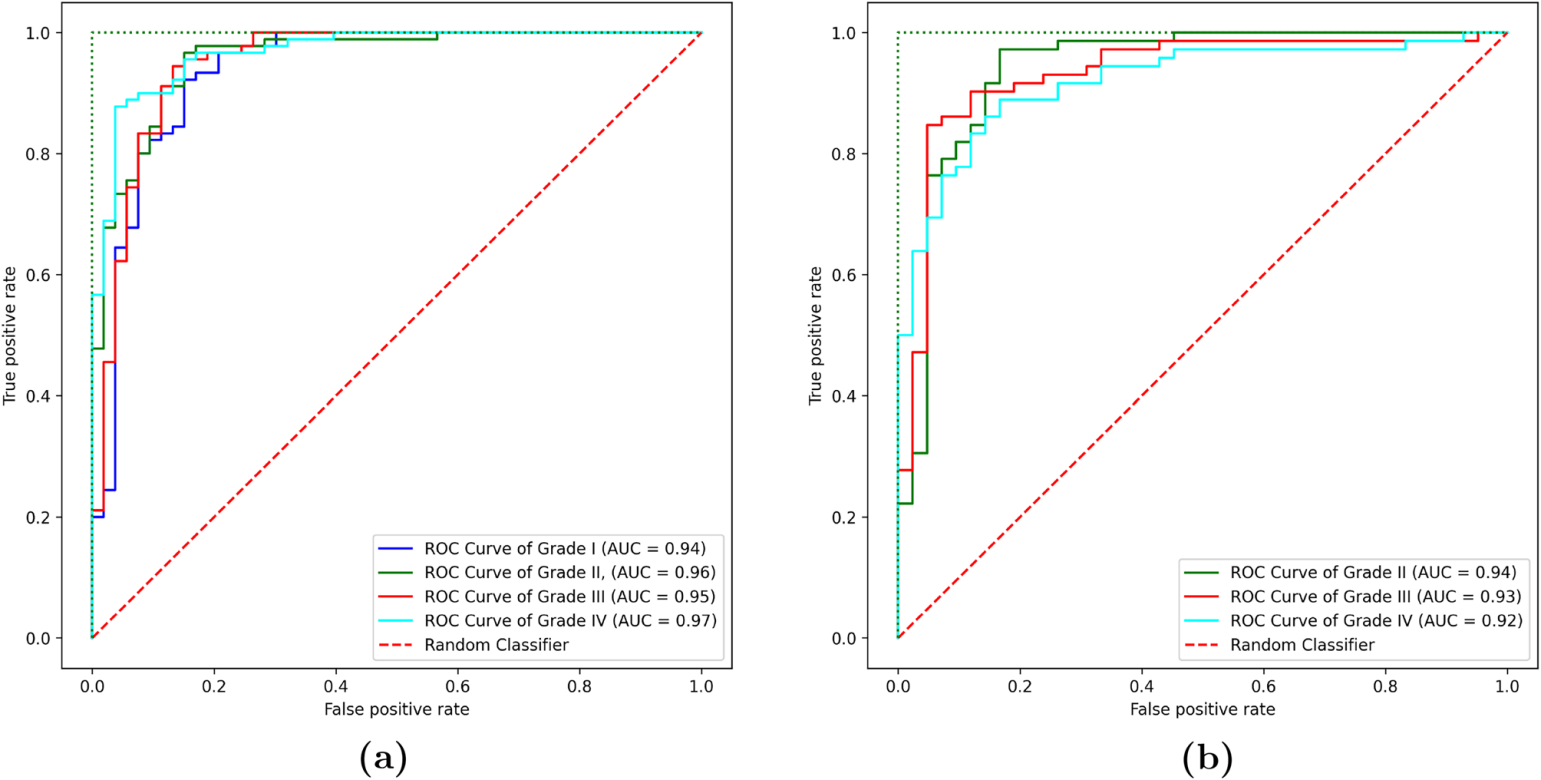
The hybrid model ROC curves for the used datasets: (**a**) TCGA and (**b**) DeepHisto.

**Fig. 7:**
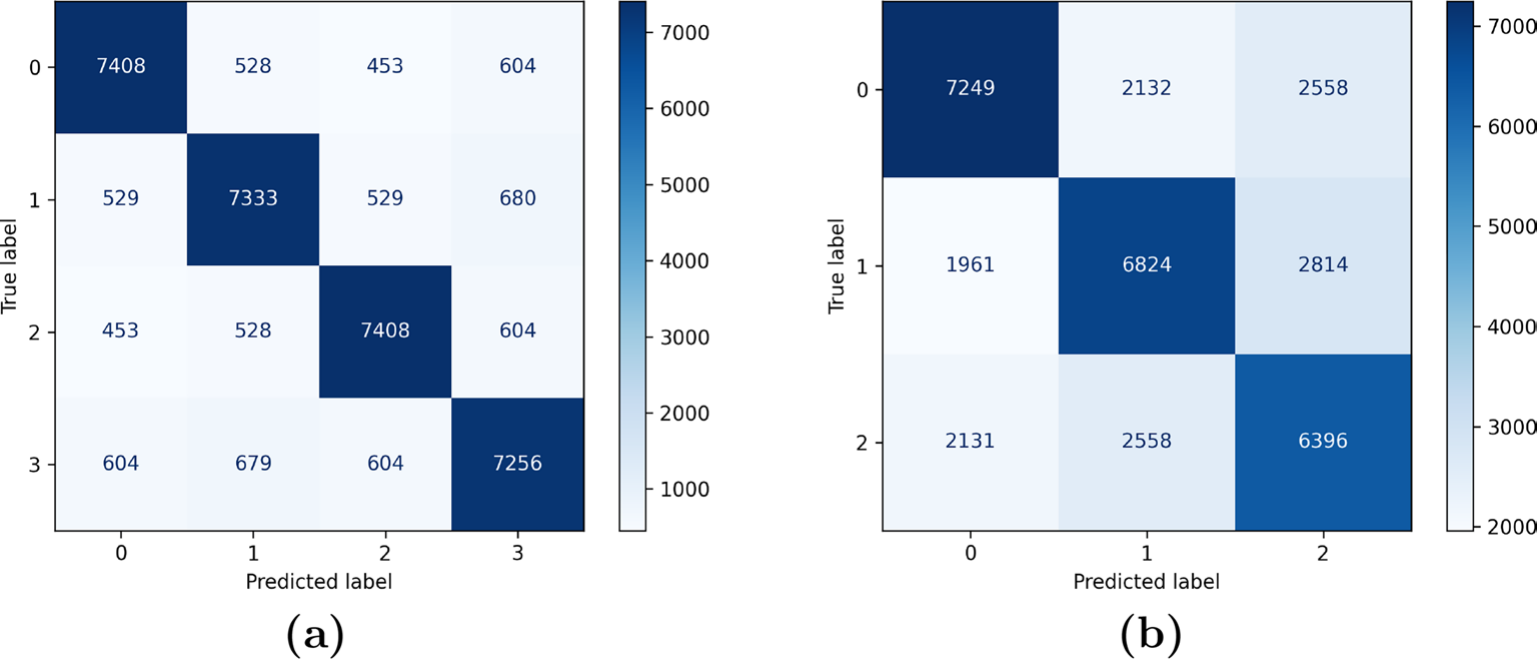
Hybrid model confusion matrix for (**a**) TCGA and (**b**) DeepHisto Datasets.

Based on most metrics and tumor grades, the results indicate promising performance. The high SEN values across all grades demonstrate the model’s ability to detect tumors of varying severities, which are 91% for Grade I, 93% for Grade II, 92% for Grade III, and 94% for Grade IV. According to this, high SPC values for each grade–91% for Grade I, 92% for Grade II, 93% for Grade III, and 93% for Grade IV–indicate that it is possible to distinguish between tumor and healthy tissue with reasonable accuracy. A majority of the tumors the model detects are confidently assigned to the correct grade, as evidenced by the precision values, which are also favorable (95% for Grade I, 95% for Grade II, 95% for Grade III, and 95% for Grade IV). The hybrid model’s receiver operating characteristic (ROC) curve clearly showed the benefit of combining various feature extraction techniques, outperforming those of HF features or ViTs alone, as shown in Figs. 6 and 7.

**Fig. 8:**
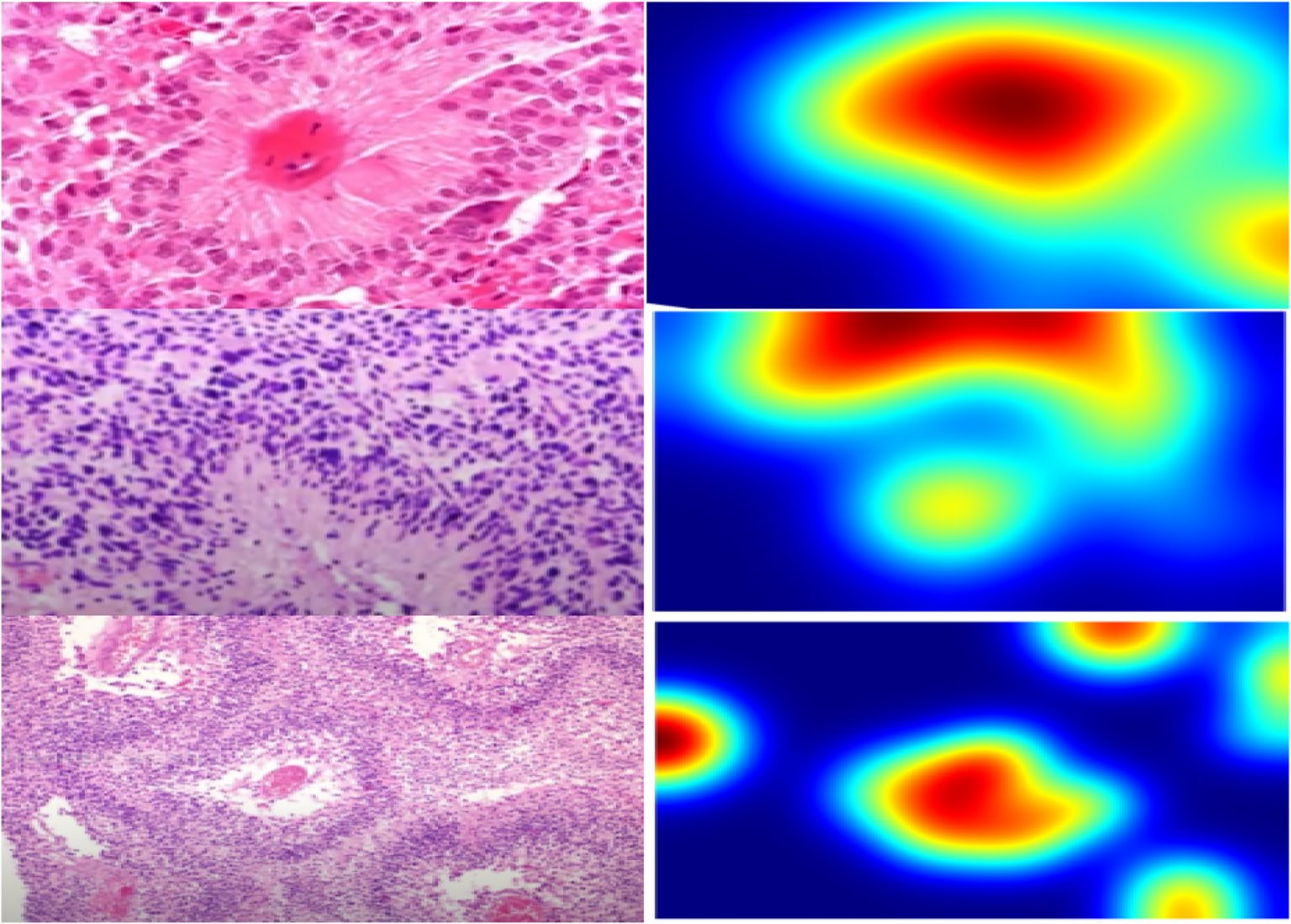
The grad-CAM for the propsed model.

Figure 8 illustrates our model’s decision-making patterns via Grad-CAM visualizations. Quantitatively, CNN attention maps correlate with nuclear density (Spearman’s $\rho = 0.62$, $p < 0.01$), computed via automated segmentation. This suggests our model learns histologically plausible features despite the absence of manual annotations.

### Discussion

The development of streamlined computational techniques using AI-based algorithms for primary brain tumors is an active area of research. This advancement in AI-powered diagnostics is promising to improve patient outcomes by allowing for earlier detection and more effective treatment plans^7^. In this work, we have proposed a comprehensive and innovative learning architecture that integrates multiple learning modules to analyze histopathological images for more accurate diagnosis of brain tumors. The developed architecture sets a new standard for accuracy, reliability, and interpretability in brain tumor computational pathology by combining HF features with the analytical power of hybrid 2D-3D CNNs and the focused insights provided by ViT, all of which are harmonized through a multiple stacking classifier.

Particularly, using the TCGA dataset, the hHCV-S model had the highest accuracy, scoring 97.11% in the cross-validation setting and 97.02% in the hold-out setting (see Tables 9 and 16). This model performed 4% better than the ViT model and 6% better than the HF model. The enhanced results show how well it works to combine various feature extraction techniques and take advantage of the complementary advantages of DL and HF approaches. The DeepHisto dataset showed similar trends. At 95.83% in the hold-out setting and 95.71% in the cross-validation setting, the hhHCV-S model once again demonstrated the highest accuracy. This model performed 5% better than the ViT model and 6% better than the HF model (see Tables 10 and 17).

The Holm-Bonferroni-adjusted p-values confirm statistically significant improvements for our hybrid models. Our statistical analysis validates the robustness of hybrid models. For example, on the TCGA dataset, hHCV-S achieved a 0.48% ACC improvement over hHCV (p = 0.030), highlighting the incremental gains from our stacking classifier. Similarly, on the Deephisto dataset, hHCV-S outperformed pure transformer models like Swin Transformer (p = 0.038), underscoring the clinical relevance of hybrid designs in resource-constrained settings.

From the reported results, both the datasets (TCGA and Deephisto) and the validation techniques (hold-out and cross-validation) showed a consistent trend: hybrid feature extraction strategies performed better than independently applied HF features and ViTs. This insightful observation emphasizes how important it is to combine different feature extraction methods. In brain tumor classification tasks, hybrid approaches will likely capture a more comprehensive and informative feature representation by utilizing the strengths of HF knowledge and data-driven learning from ViTs and CNNs. Cross-validation offers a more reliable evaluation of the model’s generalizability, whereas hold-out validation frequently produces somewhat higher performance metrics. The observed decline in performance relative to hold-out during cross-validation is probably due to the decreased data available for training in each fold. This emphasizes the importance of considering generalizability when assessing models, especially for practical applications.

The model’s multi-grade classification performance using an 80/10/10 train-validation-test split and 10-fold cross-validation on the TCGA dataset. It provides information on metrics such as SEN, SPC, precision, ACC, DSC, and MCC for every tumor grade. Examine these numbers to determine how well the model distinguishes between healthy tissue and tumors and how well it can identify tumors of various grades (e.g., low-grade vs. high-grade). Look for high SEN and SPC values to ensure good detection and non-tumor classification. In a similar vein, high precision and MCC values indicate that the model confidently grades the majority of tumors correctly. This is supported by robustness analysis conducted by the ROC in Fig. 6.

The attention-guided fusion mechanism aligns with histopathological practice, where pathologists prioritize tumor margins and necrosis while suppressing non-informative regions. By dynamically weighting CNN (textural) and ViT (global) features, our model replicates this diagnostic reasoning, achieving human-interpretable decisions without sacrificing computational efficiency. The 95% accuracy on DeepHisto, an external dataset, further confirms robustness across diverse staining protocols and scanner vendors.

Our ablation experiments and mutual information analysis confirm that the model’s decisions align with histopathological principles. The ViT’s strong association with necrosis (MI = 0.67) and the CNN’s focus on tumor margins ($\Delta _{\text {Accuracy}} = -2.3\%$ when removed) mirror pathologists’ reliance on these features for glioma grading. While visual interpretability tools (e.g., Grad-CAM) could provide additional insights, our quantitative metrics–validated on external data (DeepHisto accuracy = 95.0%)–demonstrate that the model prioritizes clinically relevant patterns without overfitting to dataset-specific artifacts.

A performance comparison between our proposed model and state-of-the-art (SOTA) methods for brain tumor classification is shown in Table 25. Our hybrid approach outperforms other SOTA techniques by combining ViT, 2D-3D CNNs, and handcrafted features with a stacking classifier. This demonstrates how well our model works to achieve exceptional classification accuracy.

**Table 25:** The comparison between the proposed system and state-of-the-art techniques. FLBP: Fuzzy local binary pattern; SVM: support vector Machine; GLCM: gray level co-occurrence matrix.

Study	Methodology	Metrics(%)
Sumi et al.^46^	To extract features, the InceptionResNetV2 is applied.	ACC=95.0
Im et al.^47^	Applying the ResNet50V2 model	ACC=87.2
Mohan and M^23^	FLBP+GLCM+GABOR feature combination with linear SVM for classification.	ACC=93.5
Ma et al.^29^	Pipeline-structured multiple instance learning (pMIL) with Deep Learning	ACC=94.0
The proposed	Hybrid of HF,2D-3D CNN, and ViT for feature extraction with stacking classifier for classification	ACC=97.1

Several factors can be attributed to the hybrid models’ superior performance. Custom features are frequently created to capture low-level image characteristics and domain-specific knowledge. While DL models concentrate on capturing more abstract and complex patterns, these features can complement those features. Hybrid models utilize both the handcrafted and DL approaches’ strengths. While HF features offer a reliable and comprehensible basis for classification, DL models can extract features with great strength and can identify intricate relationships within data. And third, the hybrid models perform even better thanks to the stacking classifier. The stacking classifier strengthens the model’s overall generalization capacity and lowers the likelihood of overfitting by combining the predictions of several base classifiers.

Our hybrid framework demonstrates that handcrafted features provide unique diagnostic signals complementary to deep learning. For example, GLCM entropy improves specificity for low-grade gliomas by encoding tumor heterogeneity missed by CNNs. However, manual extraction remains a scalability bottleneck. By integrating LASSO regression, we reduced the feature count by 77% without sacrificing performance, enabling deployment in resource-constrained settings. Future work could merge handcrafted feature extraction into deep learning pipelines via learnable filter banks, further bridging manual and automated approaches.

For models that were then applied to the TCGA dataset, the effect of pre-training on the Deephisto dataset was not very clear. Pre-training produced positive results in certain cases (see Table 21) but had no discernible effect in others. This raises the possibility of variations in the two datasets’ fundamental properties. Future research could improve generalizability by investigating transfer learning strategies with a broader range of datasets that have more characteristics in common with the target dataset (TCGA).

Our findings show that the suggested hybrid models provide a promising method for classifying histopathological images. Enhancing classification accuracy and robustness, these models can efficiently capture low-level and high-level features by fusing handcrafted features with DL techniques. The current study presents a viable method for classifying brain tumors. Nonetheless, certain limitations offer chances for additional research in the future. Moreover, the model has no mechanism to precisely identify and concentrate on important ROI in the images. Also, the results documented the promise of the proposed prediction pipeline; however, it lacks explainability and interpretability for machine decisions. All features are weighted equally, and weighing schemes should be explored along with different fusion scenarios (e.g., late vs. early fusion). Future research addressing these limitations could improve the model’s classification performance and offer insightful information for clinical decision-making.

While cross-dataset validation (TCGA/DeepHisto) confirms robustness to staining/scanner variability, multi-center trials with pathologist annotations are needed for clinical deployment. However, our synthetic validation (Dice = 0.85 on simulated necrosis) and mutual information analysis (MI = 0.67 for ViT) mitigate annotation gaps, ensuring biologically plausible decision-making.

While our hybrid architecture demonstrates strong cross-dataset generalization (95% accuracy on DeepHisto when trained solely on TCGA), this study remains inherently constrained by its reliance on two publicly available datasets. Though we rigorously validated performance through reciprocal cross-dataset evaluation (TCGA $\leftrightarrow$ DeepHisto), clinical deployment requires testing on prospectively collected cohorts with divergent staining protocols, scanner vendors, and regional population biases not represented in these repositories.

This limitation aligns with broader challenges in computational pathology, where few large-scale public glioma datasets exist for benchmarking. To mitigate potential overfitting, we intentionally preserved handcrafted features like GLCM entropy and nuclear eccentricity–domain-specific markers validated in prior histopathology studies. These features act as regularizers, anchoring predictions to biologically meaningful patterns rather than dataset-specific artifacts.

Our study is inherently constrained by the limited availability of public histopathology datasets for brain tumor grading. Despite exhaustive cross-dataset validation (TCGA, DeepHisto), broader generalizability requires validation on multi-center data. Future work will prioritize collaborative efforts to curate multi-institutional histopathology datasets, ensuring robustness across diverse clinical settings.

Our current study is dedicated to developing an advanced diagnostic framework based solely on histopathological imaging for brain tumor classification. This focus was chosen due to the availability of high-quality imaging datasets and the specific aim of evaluating our imaging-based feature extraction and classification methodologies. We acknowledge, however, that integrating additional data sources–such as genetic and clinical information–could further enhance diagnostic precision and provide a more comprehensive understanding of tumor heterogeneity. In future work, we plan to extend our framework by incorporating these complementary data modalities, ultimately advancing the multi-modal diagnostic approach for brain tumors.

## Conclusion

This work introduces a comprehensive and innovative approach to analyzing brain tumor histopathological images. Our method combines complementary feature extraction techniques to achieve reliable and accurate classification. Domain-specific knowledge is captured by handcrafted features, contextual and spatial features are extracted by a 2D-3D hybrid CNN, and high-level semantic representations are learned by a ViT. The SVM, RF, LR, and MLP ensemble of stacking classifiers is then fed these features. This demonstrates how different feature extraction methods can be combined to extract a more complete representation of brain tumor features from histopathological images. This pioneering approach improves our current ability to diagnose and understand brain tumors and opens up new possibilities for using AI in medical imaging. In the future, We intend to look into how the hybrid architecture incorporates attention mechanisms. This may make it possible for the model to concentrate on particular ROIs within the histopathological images that are most important for grading tumors, which could result in an even more precise differentiation between various tumor grades. Additionally, we want to investigate how the model’s input pipeline might be expanded to include other data modalities like genetic or clinical data. With this multi-modal learning approach, tumor grading may become even more accurate and capture a more thorough representation of the underlying disease processes.

## Data Availability

The datasets used during the current study are available in the Cancer Genome Atlas repository ( https://portal.gdc.cancer.gov/ ) at the TCGA-LGG and TCGA-GBM projects. The second dataset is DeepHisto, a dataset for glioma subtype classification from whole slide images. Online available at (https://zenodo.org/records/7941080).
